# Changes in Spanish lifestyle and dietary habits during the COVID-19 lockdown

**DOI:** 10.1007/s00394-022-02814-1

**Published:** 2022-02-07

**Authors:** Rosa Casas, Blanca Raidó-Quintana, Ana María Ruiz-León, Sara Castro-Barquero, Isabel Bertomeu, Jordi Gonzalez-Juste, Marta Campolier, Ramon Estruch

**Affiliations:** 1 Department of Internal Medicine, Hospital Clinic, Institut d’Investigació Biomèdica August Pi i Sunyer (IDIBAPS), University of Barcelona, Villarroel, 170, 08036 Barcelona, Spain; 2 CIBER 06/03: Fisiopatología de la Obesidad y la Nutrición, Instituto de Salud Carlos III, 28029 Madrid, Spain; 3 Mediterranean Diet Foundation, Barcelona, Spain; 4 Nuffield Department of Orthopaedic, Rheumatology and Musculoskeletal Sciences, Oxford Trauma and Emergency Care, Kadoorie Research Centre, University of Oxford, Oxford, UK

**Keywords:** Mediterranean diet, Physical activity, Nutrition, COVID-19, Lockdowns’ dietary behaviours, Public health

## Abstract

**Purpose:**

The COVID-2019 pandemic forced many governments to declare the “to stay at home” which encouraged social distancing and isolation among citizens. The aim of this study was to assess the dietary and lifestyle habit changes that occurred during home confinement in Spain.

**Methods:**

An European online survey was launched in April 2020. This included 70 questions on sociodemographic characteristics, lifestyle, dietary habits, including key Mediterranean diet (MedDiet) foods. A total of 945 Spanish adults from 1268 European that completed the online survey were included in the analysis.

**Results:**

Most of the Spanish participants adopted healthier dietary habits during home lockdown, which was translated to a higher MedDiet adherence. However, a negative impact on physical activity levels, sleep quality or smoking rates was observed. Low MedDiet adherence was associated with a higher risk of weight gain (OR = 1.53, CI 1.1–2.1; *p* = 0.016), while no snacking between meals reduced the risk by 80% (OR = 0.20, CI 0.09–0.45, *p* < 0.001) and eating more quantity, considering portion size, increased body weight gain risk almost sixfold more.

**Conclusion:**

To conclude, although dietary habits were improved during home lockdown, certain unhealthy behaviours (e.g. increased snacking between meals, increased food intake, and an increase in sedentary behaviour) were increased.

**Supplementary Information:**

The online version contains supplementary material available at 10.1007/s00394-022-02814-1.

## Introduction

On December 31, 2019, the World Health Organization (WHO) alerted a coronavirus (SARS-CoV-2) outbreak, which initially affected the lungs causing interstitial pneumonitis and a severe acute respiratory syndrome. It was later observed that this infection also affects many others organs and increases the risk of cardiovascular events [1]. The WHO declared a coronavirus disease 2019 (COVID-19) global pandemic on January 31, 2020 [2].

To manage the health crisis caused by the COVID-19 pandemic, various public health recommendations and governmental measures were applied. Several countries declared states of emergency, including the Spanish government on March 14, 2020 [3]. This emergency declaration caused a stockpiling of food due to the possibility of an insufficient supply in grocery stores. During the lockdown, severe mobility restrictions were applied and people were encouraged to stay at home and work remotely, with only very limited movements being allowed [4]. Kindergartens, schools, and universities were also closed, along with gyms and swimming pools [5]. Due to the “to stay at home” order, which encouraged social distancing and isolation among citizens, a sudden change in dietary and lifestyle habits occurred among the population [3].

Staying at home, working from home, and self-isolation can affect dietary habits (e.g. increasing snacking between meals, favouring eating without control, or choosing unhealthy foods to combat boredom and stress) and lifestyle-related behaviours. A reduction in physical activity (PA) levels, together with an increase in stress, fear, sadness, and anxiety, can lead to a reduction in sleep quality, as well as increased alcohol consumption and smoking [6–8]. The pandemic conditions can also increase obesity rates, which is an important predictor of increased mortality due to COVID-19 [9].

This new form of living may also have limited access to fresh foods (e.g. fruits, vegetables, and fish), causing an increase in the consumption of foods with a higher energy density and lower nutritional quality [10], including processed foods such as snacks or convenience foods. In addition, home lockdowns may have led to overeating, particularly of foods rich in sugar, as a consequence of the stressful situation derived from boredom and constantly hearing about COVID-19 in the media [11, 12]. The consumption of these “comfort foods” is widely associated with a higher risk of developing obesity and cardiovascular disease (CVD), thereby potentially increasing the risk of COVID-19-associated complications [13]. The restrictions imposed can also substantially affect other lifestyle aspects, such as smoking, which can aggravate severe COVID-19 symptoms [14], or alterations in the sleep–wake rhythm that are induced by obesity [15].

Following a healthy diet, such as the Mediterranean diet (MedDiet), has been linked to a lower risk for all-cause and cardiovascular mortality, myocardial infarction, stroke, type-2 diabetes mellitus, cancer, Alzheimer’s disease, obesity, and CVD risk factors [16, 17].

Evidence suggests that several behavioural changes have occurred during COVID-19 outbreak. Increases in several side effects related to lifestyle were observed, including increased sleepiness, a higher consumption of ultra-processed foods, and increased sedentary behaviour. Thus, the main objective of this study was to evaluate the dietary and lifestyle habits changes that occurred during home confinement in several European countries, with a particular emphasis on the MedDiet basin countries. In addition, we aimed to analyse the risk of weight gain in participants according to their dietary pattern and lifestyle (e.g. quality of sleep and levels of PA). To this end, a self-administered online survey was distributed to adult citizens from seven European countries through social media, instant messaging platforms, and email to assess the pandemic-induced changes in their dietary and lifestyle behaviour. As consequence of several limitations, explained in material and methods, only Spanish participant data was included in the present analysis.

## Materials and methods

### Study design and participants

This cross-sectional study among European adults was designed by the Mediterranean Diet Foundation (https://dietamediterranea.com) based on the framework of Interreg Med-MD.net (https://mdnet.interreg-med.eu). The study aimed to gain a better understanding of the effects of the pandemic on dietary patterns, lifestyle factors, body weight and other CVD risk factors. The online questionnaire was made available to European adults (aged >18 years) for 40 days, from April 23, 2020 to June 2, 2020.

The survey included 70 questions on sociodemographic characteristics, lifestyle, dietary habits, and the consumption of key MedDiet foods, such as olive oil, nuts, wine, fruits and vegetables. The online survey was self-administered and divided in different sections. The sociodemographic section included questions on age, sex, age, habitual housing, place of residence, marital status, educational level, children or elderly in their care, work status, and the number of days confined, among others. Regarding diet, the questions related to dietary habits, such as snacking, skipping meals, meal frequency, cooking techniques, purchase habits, and food intake (all related to the MedDiet pattern). The questions on dietary habits also included a 14-item validated MedDiet adherence score (MEDAS) instrument, where scores could range from 0 to 14 points. This instrument has been shown to be a simple, reliable, and effective tool to assess adherence to the MedDiet [18, 19]. Other questions asked about the daily consumption of certain foods (e.g. sweetened beverages and pastries), the frequency of intake of unhealthy foods (e.g. sweets and type of snacks), the frequency of eating, and the number of meals per day. Questions were also asked regarding the intake of water, alcohol, juices and soft drinks, and cooking techniques used. Lifestyle characteristics measured included physical activity (PA), sleep time and quality of sleep, smoking habits, sociability (time shared with family), and changes in body weight. All questions were aimed to be answered during the lockdown period. The full version of the questionnaire is available as Appendix 1.

A total of 1268 European adults completed the online survey (945 Spanish and 323 from other European countries). Although the online survey was primarily completed by adults from seven MedDiet basin countries, questionnaires were also received from other European countries, including Italy (8.3%), Bosnia and Herzegovina (3.2%), Greece (3.2%), Portugal (1.8%), Albania (1.7%), Croatia (1.4%), Slovenia (1.2%), France (0.8%), the United Kingdom (0.7%), Germany (0.6%), Cyprus (0.3%), Andorra (0.2%), Austria (0.2%), the Netherlands (0.2%), Poland (0.2%), Romania (0.2%), Russia (0.2%), Belgium (0.1%), the Czech Republic, (0.1%), Hungary (0.1%), Luxemburg (0.1%) and Sweden (0.1%).

It must be noted that the group of 323 people from other European countries was smaller and less homogeneous compared to the Spanish group. The European group was very culturally diverse and likely adhered less to a MedDiet. On the other hand, the classic eating pattern in Spain is the MedDiet. This term, coined in the mid-twentieth century, refers to much more than mere ingredients or a particular type of eating habit. The term also refers to lifestyle, including behavioural habits, values, and social structure. Given that the characteristics of local or national lockdowns imposed by governments differed across the European countries, it could be challenging to compare the results across countries or regions. However, in Spain, similar public health recommendations and governmental measures were applied throughout the Spanish territory during the COVID-19 pandemic. Thus, we decided to focus on the Spanish group, as it was much larger and more homogeneous than the European group.

### Data privacy and consent for participation

This study was conducted in compliance with EU regulation 2016/679 of the European parliament and the council of April 27, 2016 on the protection of individuals regarding the treatment of their personal data. The study also complies with organic law 3/2018 of December 5th regarding the protection of personal data and guarantees of digital rights. No informed written consent was requested because the online survey was anonymous and no personal data were collected. Therefore, the online survey study did not require approval by an ethics committee.

Although participation was voluntary, all participants were informed of the aim of the study and were asked for permission to add their data to a European database that could be used by other research groups to conduct similar surveys in other countries. In addition, the participants were able to leave the online survey at any time before clicking the “submit” button and sending their answers. In this case, their data were not saved.

Each completed questionnaire was submitted to the EUSurvey online platform and the final database was downloaded as a Microsoft Excel sheet.

### Procedure and promotion

To cover all of Europe and reach the maximum number of potential participants, the electronic survey was uploaded to the EUSurvey online platform (https://ec.europa.eu/eusurvey/). Direct links to the electronic survey were distributed using a wide range of online services, including email, LinkedIn, Facebook, Twitter and WhatsApp. The electronic survey was also translated and administered in several different languages, including Spanish, English, Greek, Italian, Portuguese, Slovenian, Croatian and Albanian. The EUSurvey online platform has a tool that allows for automatic translation into all official languages of the European Union while leaving the original meaning unchanged. There was not a back-translated Spanish version.

Thirteen partners of Interreg Med-MD.net helped to promote and disseminate the electronic survey: Campania Regio (Italy); MeditBio- Centre for Mediterranean Bioresources and Food, University of Algarve (Portugal); RERA S.D. for Coordination and Development of Split Dalmatia County (Croatia); Directorate of Environment and Spatial Planning, Region of Crete (Greece); Faculty of Agriculture and Food Technology, University of Mostar (Bosnia and Herzegovina); Mediterranean Diet Foundation (Spain); Official Chamber of Commerce, Industry and Shipping of Seville (Spain); Directorate General, Knowledge, Labour and Enterprise Economy, Emilia Romagna Region (Italy); Institute for Comprehensive Development Solutions, eZAVOD (Slovenia); Municipality of Caltanissetta (Italy); Association of Albanian Municipalities (Albania); PRODECA (Spain); Troodos Development Company Ltd. (Cyprus).

### Statistical analyses

For descriptive statistics, the mean ± SD was used for continuous variables and the number and percentage (%) for categorical variables. All of the collected variables were assessed and the participants were split into the following groups according to age: <33 years (first quartile), 33–43 years (second quartile), 44–52 years (third quartile) and ≥53 years (fourth quartile). The Shapiro–Wilk test was used to evaluate variable distributions. Student’s *t* tests (for continuous normally distributed data), Kruskal–Wallis tests (for non-normally distributed data), or Chi-squared tests (for categorical data) were used for comparisons. *p* values were corrected for multiple comparisons using the Holm–Bonferroni’s method as a post hoc test.

First, participants were divided into in three groups according to their MedDiet score [low adherence (<8), medium adherence (8–9) or high adherence (≥10)] and the association with body weight changes was assessed. Second, the quantity (increase vs. decrease of food intake) and quality (high vs. low dietary quality) of the meals was evaluated and participants were divided into the following four groups: (a) decrease in quantity and high quality; (b) decrease in quantity and low quality; (c) increase in quantity and low quality; (d) increase in quantity and high quality. Third, PA was evaluated by dividing the participants into different categories according to changes in PA levels compared to those before lockdown: (a) as before (no changes in PA level); (b) increased PA; (c) decreased PA; (d) no PA. In addition, the participants were classified according to the time spent doing PA (daily between 0 and 60 min or >60 min): (a) 5–7 days a week; (b) 3–4 times a week; (c) once to twice a week; (d) once a week; (e) no PA. Finally, sleep quality and hours of sleep were also assessed. To evaluate sleep quality, the participants were divided into three groups according to changes in sleep quality compared to before the lockdown: (a) no changes; (b) better; (c) worse. Moreover, the hours of sleep were quantified and used to divide the participants into five groups: (a) < 5 h; (b) 5–6 h; (c) 6–7 h; (d) 7–8 h; (e) > 8 h.

Body weight change was treated as a binary variable, where those participants who showed weight loss or body weight maintenance were the reference category set to zero and those who showed body weight gain were set to one. To evaluate the influence of the analysed variables (MedDiet adherence, specific food intake, dietary habits, and lifestyle) on weight gain, logistic regression models were used.

Dummy variables were created and two models were applied to evaluate: (a) a univariate regression model (model 1) and (b) a multivariate-adjusted model (model 2). Model 2 was adjusted by potential confounders, including sex (men and women), educational level (postgraduate, university, high school, primary, and other studies), housing during lockdown (with or without children, with elderly, children and elderly, residence or shared flat) and PA level (high, medium, low). Data were expressed using odds ratios (ORs) and their corresponding 95% confidence intervals (CIs).

Results were considered significant at a *p* < 0.05. Statistical analyses were performed using SPSS ver. 22.0 (IBM, Chicago, IL, USA).

## Results

### Sociodemographic characteristics

A total of 945 Spanish adults completed the online survey. Table 1 shows the main sociodemographic characteristics of the participants.

**Table 1:** Main characteristics of the participants

	Spanish (*N* = 945)	< 33 y. *N* = 243	33–44 y. *N* = 248	44–53 y. *N* = 218	> 53 y. *N* = 236	*P*¥
Gender						0.001
Women	273 (28.9)*	181 (74.5)	190 (76.6)	157 (72.0)	141 (59.7)	
Men	669 (70.8)	62 (25.5)	58 (23.4)	61 (28.0)	95 (40.3)	
Smoking status						0.032
Current smoking	180 (19.0)	60.0 (24.7)	40.0 (16.1)	44.0 (20.2)	36.0 (15.3)	
Habitual residence					0.018	
Urban	842 (89.1)	217 (89.3)	227 (91.5)	182 (83.5)	216 (91.5)	
Rural	103 (10.9)	26 (10.7)	21 (8.5)	36 (16.5)	20 (8.5)	
Living during confinement						< 0.001
With children	475 (50.3)	63 (25.9)	142 (57.3)	158 (72.5)	112 (47.5)	
Without children	257 (27.2)	98 (40.3)	57 (23.0)	25 (11.5)	77 (32.6)	
Shared flat	58 (6.1)	40 (16.5)	6 (2.4)	3 (1.4)	0 (0.0)	
Alone	8 (0.8)	15 (6.2)	29 (11.7)	15 (6.9)	32 (13.6)	
With parents or other relatives	49 (5.2)	24 (9.9)	10 (4.0)	11 (5.0)	13 (5.5)	
Residence	1 (0.1)	1 (0.4)	0 (0.0)	0 (0.0)	0 (0.0)	
Others^a^	91 (9.6)	0 (0.0)	3 (1.2)	3 (1.4)	2 (0.8)	
Marital status						< 0.001
Married	450 (47.6)	12 (4.9)	120 (48.4)	150 (68.8)	168 (71.2)	
Domestic partner	160 (16.9)	70 (28.8)	56 (22.6)	20 (9.2)	14 (5.9)	
Single	262 (27.7)	157 (64.6)	57 (23.0)	26 (11.9)	22 (9.3)	
Widower	5 (0.5)	0 (0.0)	0 (0.0)	1 (0.5)	4 (1.7)	
Divorced	58 (6.1)	0 (0.0)	12 (4.8)	19 (8.7)	27 (11.4)	
Children in care						< 0.001
Yes	533 (56.4)	11 (4.5)	151 (60.9)	169 (77.5)	202 (85.6)	
No	412 (43.6)	232 (95.5)	97 (39.1)	49 (22.5)	34 (14.4)	
Educational level						
Postgraduate	287 (30.4)	88 (36.2)	85 (34.3)	54 (24.8)	60 (25.4)	
University	379 (40.1)	79 (32.5)	113 (45.6)	93 (42.7)	94 (39.8)	
High school	233 (24.7)	68 (2.9)	43 (4.8)	61 (7.3)	61 (6.8)	
Primary	29 (3.1)	7 (25.1)	2 (12.5)	1 (20.6)	5 (19.1)	
Other	15 (1.6)	0 (2.9)	4 (0.8)	9 (0.5)	16 (2.1)	
No studies	2 (0.2)	1 (0.0)	1 (1.6)	0 (4.1)	0 (6.8)	
Current employment						< 0.001
Employee	559 (59.2)	105 (43.2)	183 (73.8)	150 (68.8)	121 (51.3)	
Independent	107 (11.3)	13 (5.3)	27 (10.9)	33 (15.1)	34 (14.4)	
ERTE^b^	54 (5.7)	20 (8.2)	16 (6.5)	12 (5.5)	6 (2.5)	
Student	88 (9.3)	85 (35.0)	1 (0.4)	2 (0.9)	0 (0.0)	
Retiree	69 (7.3)	0 (0.0)	2 (0.8)	2 (0.9)	65 (27.5)	
Subsidiary	66 (7.0)	19 (7.8)	19 (7.7)	19 (8.7)	9 (3.8)	
Current employment/education form						< 0.001
Online	469 (49.6)	142 (58.4)	128 (51.6)	108 (49.5)	91 (38.6)	
Face-to-face	144 (15.2)	23 (9.5)	45 (18.1)	40 (18.3)	36 (15.3)	
Mix^c^	85 (9.0)	24 (9.9)	22 (8.9)	23 (10.6)	16 (6.8)	
Other forms^d^	247 (26.1)	41 (16.9)	34 (13.7)	27 (12.4)	75 (31.8)	
Family economy					0.075	
High income	96 (10.2)	15 (6.2)	30 (12.1)	24 (11.0)	27 (11.4)	
Medium income	664 (70.3)	166 (68.3)	176 (71.0)	154 (70.6)	168 (71.2)	
Low income	185 (19.6)	62 (25.5)	42 (16.9)	40 (18.3)	41 (17.4)	

	Spanish (*N* = 945)					
Age (years)	43.4 ± 13.4*	25.7 ± 4.5	40.1 ± 3.2	48.5 ± 2.5	60.4 ± 6.0	< 0.001
Days^e^	43.4 ± 10.8	45.5 ± 11.5	43.3 ± 10.1	41.6 ± 10.8	42.9 ± 10.7	0.001

^*^Values are mean ± SD or *n* (%) as appropriate. ¥ANOVA-one factor was used for continuous variables and *χ*^2^-test for categorical variable

^a^Others: with children and parents or other relatives’ parents or other relatives

^b^*ERTE* temporary employment regulation file

^c^Mix: combines face-to-face and online

^d^Other forms: retiree, subsidiary, unemployed and total-ERTE

^e^Days of confinement

On average, participants were 43.4 ± 13.4 years old and spent 43.4 ± 10.8 days in lockdown. Nearly, three-quarters of the participants were female (71%), 89% lived in urban areas, 51% had children in their care, and 48% were married. In addition, the majority of participants were employed (59.2%) and defined their income level as medium (70%). 5.2% of the participants shared a flat, 9.6% lived alone, and 0.1% lived in residences. Most of the participants had a university education or postgraduate degree (70.5%), while 29.6% had a high school, primary or another type of education, and 0.1% did not study.

### Dietary and lifestyle changes during lockdown

Dietary and lifestyle changes are shown in Supplementary Table 1. Regarding body weight, 46% of the participants maintained their weight, 33% gained weight, and 12% lost weight. Regarding dietary habits, 34.5% of the participants reported an increase in the quantity of food intake and 41% reported a reduction in snacking, compared to before lockdown. The preferences for snacks were chocolate (43%), fruits and vegetables (41%), and raw nuts (30%). The participants also showed a low tendency to skip meals (76.2%). In addition, 19.2% of the participants reported an increase in eating and an improvement in the quality of foods, and 15% reported a reduction in the number of meals and an improvement in food quality. Although most Spanish participants preferred to consume local products, both fresh and frozen foods were also eaten. Moreover, most of the participants reported an increase in the time spent cooking and did not increase the consumption of fast or convenience foods. Among the culinary techniques used, griddle (78.7%), roast or oven (68.4%), boiled or steamed (59%), and casseroles or stews (55.7%) were preferred by the participants. With regard to sociability, Spanish participants reported an increase in the number of meals shared with family members (50%) compared to 34.5% of participants prior to lockdown.

PA levels decreased by 63.1% in the older participants group in comparison to the youngest group (44%). It should be highlighted that 30.6% of the participants exercised about 30–60 min almost every day, but only 8.8% exercised for more than 1 h a day.

Nearly, half of the participants reported sleeping the same compared to before confinement, with a minimum of 7 h per day.

Finally, 51.1% were non-smokers and 19% were smokers. For the current smokers, a quarter of them (25%) reported that they smoked less.

### MedDiet adherence during lockdown

MedDiet adherence during lockdown according to age groups is shown in Table 2. Spanish participants showed a MEDAS MedDiet Adherence Score [20] of 8.9 ± 1.6.

**Table 2:** MedDiet adherence Score assessed according to age during the COVID-19 lockdown

	Spanish (*N* = 945)	SD
	Mean	
Age, years		
<33 y	8.7	1.6
33–44 y	8.7	1.6
44–53 y	8.9	1.6
>53 y	9.4	1.6

y: year

Supplementary Table 2 shows key food intake and MedDiet pattern changes during the COVID-19 lockdown. Briefly, most of the participants consumed 1–2 pieces a day of fruits, vegetables and dairy products. In addition, they ate 1–2 servings of legumes a week. Most of the participants (≥40%) also consumed red and lean meat, as well as fish and seafood, 2–3 times a week, while 35% consumed these items less than once a week. In the case of *sofrito*, a traditional MedDiet recipe based on extra virgin olive oil, tomato, garlic and onion, 68.8% of the participants reported a low consumption (≤1 serving a week).

For beverages, 45.2% of participants reported drinking less than 5 glasses of water per day, mainly older people. For alcoholic beverages, participants aged 33–53 years showed a higher consumption of wine and beer in comparison to spirits and liquors. Most of the Spanish participants did not drink sweetened beverages or juices (fresh or commercial).

### Associations between eating, lifestyle habits and body weight changes during lockdown

As shown in Tables 3 and 4, those participants with a lower MedDiet adherence had a higher risk (53% more) of weight gain (OR = 1.53, CI 1.1–2.1, *p* = 0.016). In fact, this risk was higher (twofold more) than participants aged 34–44 or those ≥53 years. No snacking between meals reduced the risk by 80% (OR = 0.20, CI 0.09–0.45, *p* < 0.001) and eating a greater quantity of food increased the body weight gain risk almost sixfold. Skipping meals was only associated with higher risk (sixfold more) in those individuals aged 44–53 years. In addition, the quantity and quality of foods consumed affected body weight gain. Increased eating and low food quality increased the risk (OR = 27.3, CI 12.4–60.2, *p* < 0.001), whereas eating more and better quality food increased the risk by 9.1% (CI 4.7–17.6, *p* < 0.001). Fast and convenience foods, as well as fried foods, were also associated with a higher risk of weight gain (OR = 3.1, CI 1.4–6.6, *p* = 0.004; OR = 2.5, CI 1.4–4.5, *p* = 0.001, respectively).

**Table 3:** Associations between eating and lifestyle habits and weight gain during the COVID-19 lockdown

	Crude model^a^	Adjusted model^b^
	OR (95% CI)	OR (95% CI)
MeDiet Score	*N* = 294 (31.1%)	
High	**Ref.**	**Ref.**
Medium	1.37 (0.93–2)	1.42 (0.95–2.12)
Low	**1.66 (1.2–2.28)**	**1.53 (1.08–2.15)**
Snacking	*N* = 312 (33%)	
Unknown	**Ref.**	**Ref.**
No	**0.23 (0.11–0.48)**	**0.2 (0.09–0.45)**
Yes	0.92 (0.44–1.91)	0.81 (0.37–1.8)
Skip meals	*N* = 312 (33%)	
As before	**Ref.**	**Ref.**
No	0.89 (0.63–1.27)	0.94 (0.65–1.37)
Yes	1.01 (0.53–1.92)	0.89 (0.46–1.75)
Eat more	*N* = 200 (21.2%)	
As before	**Ref.**	**Ref.**
No	0.72 (0.48–1.1)	0.71 (0.46–1.1)
Yes	**5.83 (3.94–8.63)**	**6.14 (4.05–9.31)**
Quantity and quality	*N* = 294 (31.1%)	
Less and better	**Ref.**	**Ref.**
Less and worse	1.55 (0.4–5.95)	1.01 (0.25–4.11)
More and worse	**23.62 (11.53**–**48.38)**	**27.29 (12.36**–**60.22)**
More and better	**7.5 (4.13**–**13.62)**	**9.12 (4.73**–**17.6)**
Fast food	*N* = 312 (33%)	
As before	**Ref.**	**Ref.**
No	1.14 (0.72–1.8)	1.29 (0.8–2.08)
Yes	**2.6 (1.26**–**5.35)**	**3.09 (1.43**–**6.65)**
Fried foods	*N* = 312 (33%)	
As before	**Ref.**	**Ref.**
No	0.83 (0.56–1.22)	0.9 (0.6–1.35)
Yes	**2.3 (1.35–3.92)**	**2.54 (1.43–4.49)**
Cooking^c^	*N* = 312 (33%)	
As before	**Ref.**	**Ref.**
No	0.9 (0.56–1.46)	0.82 (0.5–1.37)
Yes	0.84 (0.6–1.19)	0.87 (0.61–1.26)
Sociability^d^	*N* = 291 (30.8%)	
As before	**Ref.**	**Ref.**
No	1.25 (0.74–2.13)	1.25 (0.69–2.25)
Yes	1.28 (0.95–1.74)	1.33 (0.96–1.84)
Physical activity	*N* = 312 (33%)	
As before	**Ref.**	**Ref.**
Higher	1 (0.57–1.76)	1 (0.57–1.78)
Lower	**3.99 (2.5–6.39)**	**4.05 (2.52–6.49)**
Never	**4.07 (2.07–7.99)**	**3.91 (1.97–7.75)**
Freq. of PA	*N* = 312 (33%)	
30–60 min		
Never	**Ref.**	**Ref.**
Every day or almost	**0.27 (0.18–0.41)**	**0.27 (0.18–0.41)**
3–4 times a week	**0.38 (0.25–0.58)**	**0.39 (0.26–0.59)**
Once–twice a week	0.69 (0.44–1.06)	0.69 (0.45–1.08)
Once a week	0.66 (0.4–1.08)	0.66 (0.4–1.07)
>60 min	*N* = 312 (33%)	
Never	**Ref.**	**Ref.**
Every day or almost	**0.34 (0.19–0.62)**	**0.33 (0.18–0.61)**
3–4 times a week	**0.38 (0.2–0.72)**	**0.38 (0.2–0.73)**
Once–twice a week	0.85 (0.54–1.33)	0.85 (0.54–1.35)
Once a week	0.7 (0.46–1.08)	0.71 (0.46–1.09)
Sleep quality	*N* = 312 (33%)	
As before	**Ref.**	**Ref.**
Worse	0.99 (0.73–1.34)	1 (0.74–1.36)
Better	0.93 (0.63–1.38)	0.92 (0.62–1.37)
Smoker	*N* = 312 (33%)	
Non-smoker	**Ref.**	**Ref.**
Former smoker	1.25 (0.92–1.71)	1.25 (0.91–1.72)
Current smoker	1.16 (0.8–1.67)	1.16 (0.79–1.68)
Smoking	*N* = 312 (33%)	
As before	**Ref.**	**Ref.**
Higher	0.89 (0.42–1.91)	0.94 (0.43–2.05)
Lower	**0.47 (0.3–0.73)**	**0.47 (0.3–0.74)**
Never	**0.48 (0.31–0.75)**	**0.48 (0.31–0.76)**
Days of confinement	*N* = 312 (33%)	
>50	**Ref.**	**Ref.**
45–50	0.73 (0.46–1.18)	0.72 (0.45–1.16)
40–45	0.96 (0.65–1.44)	0.93 (0.62–1.4)
<40	0.85 (0.56–1.3)	0.80 (0.52–1.23)

^a^Univariate regression model (model 1)

^b^Multivariate-adjusted model (model 2). Model 2 adjusted by: sex (men and women), educational level (postgraduate; university; high school; primary and other studies), housing during confinement (with or without children; with elderly; and children and elderly; residence or shared flat) and type of AP (high, medium, low)

^c^During the confinement, do you spend more time cooking?

^d^Time spent with family (eating together more often). ORs of the association between each studied variable related to eating and lifestyle habits and the weight gain change were mutually adjusted by each other. Statistically significant ORs are highlighted in bold. *MeDiet* Mediterranean diet

**Table 4:** Associations between eating and lifestyle habits and weight gain stratified by age during the COVID-19 lockdown

	≤ 33 y		34–43.9 y		44–52.9 y		≥ 53 y	
	Crude model^a^	Adjusted model^b^	Crude model^a^	Adjusted model^b^	Crude model^a^	Adjusted model^b^	Crude model^a^	Adjusted model^b^
	OR (95% CI)	OR (95% CI)	OR (95% CI)	OR (95% CI)	OR (95% CI)	OR (95% CI)	OR (95% CI)	OR (95% CI)
MedDiet Score	*N* = 52 (17.7%)		*N* = 91 (31%)		*N* = 71 (24.1%)		*N* = 80 (27.2%)	
High	**Ref.**	**Ref.**	**Ref.**	**Ref.**	**Ref.**	**Ref.**	**Ref.**	**Ref.**
Medium	1.75 (0.78–3.96)	1.38 (0.57–3.33)	0.71 (0.32–1.57)	0.81 (0.33–1.99)	1.73 (0.8–3.75)	1.68 (0.74–3.8)	1.79 (0.86–3.71)	1.69 (0.75–3.8)
Low	0.72 (0.34–1.51)	0.48 (0.21–1.13)	**2.26 (1.22–4.19)**	**2.23 (1.12–4.44)**	**2.26 (1.12–4.55)**	1.74 (0.81–3.75)	1.8 (0.96–3.37)	**2.06 (1.02–4.16)**
Snacking	*N* = 62 (19.9%)		*N* = 91 (29.2%)		*N* = 75 (24%)		*N* = 84 (26.9%)	
Unknown	**Ref.**	**Ref.**	**Ref.**	**Ref.**	**Ref.**	**Ref.**	**Ref.**	**Ref.**
No	0.48 (0.12–1.94)	0.42 (0.09–1.86)	**0.17 (0.05–0.57)**	**0.1 (0.02–0.48)**	**0.05 (0.01–0.51)**	**0.06 (0.01–0.8)**	0 (0–0)	0 (0–0)
Yes	1.73 (0.44–6.76)	1.43 (0.34–6.06)	0.89 (0.27–2.99)	0.62 (0.13–2.82)	0.32 (0.03–3)	0.39 (0.03–5.04)	0 (0–0)	0 (0–0)
Skip meals	*N* = 62 (19.9%)		*N* = 91 (29.2%)		*N* = 75 (24%)		*N* = 84 (26.9%)	
As before	**Ref.**	**Ref.**	**Ref.**	**Ref.**	**Ref.**	**Ref.**	**Ref.**	**Ref.**
No	**0.47 (0.24–0.92)**	0.54 (0.25–1.13)	1.05 (0.53–2.06)	1.21 (0.56–2.64)	2.03 (0.87–4.73)	1.97 (0.78–4.98)	0.68 (0.33–1.39)	0.84 (0.37–1.9)
Yes	0.22 (0.04–1.06)	0.22 (0.04–1.17)	2.11 (0.68–6.52)	2.17 (0.63–7.51)	**6.34 (1.48–27.22)**	**6.48 (1.3–32.32)**	0.16 (0.02–1.38)	0.10 (0.01–1.01)
Eat more	*N* = 44 (71%)		*N* * N* = 63 (69.2%)		*N* = 41 (54.7%)		*N* = 52 (61.9%)	
As before	**Ref.**	**Ref.**	**Ref.**	**Ref.**	**Ref.**	**Ref.**	**Ref.**	**Ref.**
No	1.02 (0.38–2.73)	1.09 (0.38–3.07)	0.46 (0.2–1.05)	**0.28 (0.1–0.78)**	0.61 (0.28–1.36)	0.71 (0.3–1.72)	0.81 (0.36–1.84)	0.67 (0.27–1.62)
Yes	**7.12 (3.05–16.63)**	**7.79 (3.11–19.52)**	**6.21 (2.93–13.15)**	**8.64 (3.42–21.81)**	**3.22 (1.47–7.05)**	**4.09 (1.7–9.85)**	**8.78 (3.79–20.32)**	**6.2 (2.48–15.52)**
Quantity and quality	*N* = 52 (23%)		*N* = 91 (31.1%)		*N* = 71 (23%)		*N* = 80 (23%)	
Less and better	**Ref.**	**Ref.**	**Ref.**	**Ref.**	**Ref.**	**Ref.**	**Ref.**	**Ref.**
Less and worse	0 (0–0)	0 (0–0)	4.75 (0.65–34.6)	5.45 (0.41–71.71)	6.2 (0.33–115.92)	8.13 (0.21–314.75)	0 (0–0)	0 (0–0)
More and worse	**21 (5.15–85.63)**	**43 (6–308.05)**	**69.67 (14.26–340)**	**172.09 (19.82–1494)**	**20.15 (4.65–87.3)**	**186.28 (17.06–2034)**	**12.5 (2.53–61.81)**	**9.49 (1.68–53.67)**
More and better	**4.33 (1.12–16.77)**	**6.73 (1.23–36.87)**	**8.23 (2.59–26.17)**	**19.81 (4.37–89.73)**	**7.75 (2.45–24.5)**	**29.16 (4.71–180.35)**	**11.72 (3.47–39.59)**	**12.39 (3.17–48.46)**
Fast food	*N* = 62 (19.9%)		*N* = 91 (29.2%)		*N* = 75 (24%)		*N* = 84 (26.9%)	
As before	**Ref.**	**Ref.**	**Ref.**	**Ref.**	**Ref.**	**Ref.**	**Ref.**	**Ref.**
No	1.62 (0.53–4.98)	1.76 (0.54–5.71)	0.93 (0.39–2.22)	0.96 (0.34–2.69)	1.65 (0.63–4.34)	1.85 (0.65–5.31)	0.8 (0.34–1.87)	0.69 (0.26–1.85)
Yes	3.5 (0.83–14.83)	3.83 (0.84–17.49)	1.94 (0.49–7.64)	2.76 (0.54–14.18)	**11.08 (1.8–68.4)**	**20.21 (2.47–165)**	1.12 (0.21–6.14)	0.77 (0.13–4.56)
Fried foods	*N* = 62 (19.9%)		*N* = 91 (29.2%)		*N* = 75 (24%)		*N* = 84 (26.9%)	
As before	**Ref.**	**Ref.**	**Ref.**	**Ref.**	**Ref.**	**Ref.**	**Ref.**	**Ref.**
No	0.83 (0.37–1.85)	1.09 (0.44–2.66)	0.77 (0.37–1.61)	1.15 (0.49–2.69)	0.57 (0.26–1.27)	0.54 (0.23–1.3)	1.14 (0.52–2.49)	0.86 (0.34–2.16)
Yes	2.57 (0.9–7.36)	**3.19 (0.97–10.48)**	**3.77 (1.39–10.22)**	**4.88 (1.55–15.35)**	2.05 (0.62–6.76)	2.25 (0.59–8.61)	1 (0.3–3.38)	0.69 (0.18–2.65)
Cooking^c^	*N* = 62 (19.9%)		*N* = 91 (29.2%)		*N* = 75 (24%)		*N* = 84 (26.9%)	
As before	**Ref.**	**Ref.**	**Ref.**	**Ref.**	**Ref.**	**Ref.**	**Ref.**	**Ref.**
No	0.48 (0.14–1.7)	0.48 (0.13–1.84)	1.27 (0.46–3.5)	1.84 (0.57–5.97)	0.78 (0.28–2.18)	0.72 (0.23–2.24)	0.97 (0.43–2.21)	1.01 (0.4–2.57)
Yes	0.92 (0.44–1.95)	0.98 (0.44–2.2)	0.72 (0.35–1.46)	1.03 (0.45–2.37)	0.96 (0.48–1.91)	0.86 (0.4–1.85)	0.82 (0.42–1.58)	0.84 (0.4–1.76)
Sociability^d^	*N* = 57 (23%)		*N* = 87 (31.1%)		*N* = 69 (23%)		*N* = 78 (23%)	
As before	**Ref.**	**Ref.**	**Ref.**	**Ref.**	**Ref.**	**Ref.**	**Ref.**	**Ref.**
No	1.59 (0.58–4.35)	1.36 (0.44–4.15)	0.49 (0.17–1.39)	0.46 (0.13–1.62)	1.46 (0.41–5.19)	1.16 (0.26–5.13)	2.69 (0.9–8.07)	2.18 (0.6–7.89)
Yes	1.4 (0.73–2.7)	1.29 (0.64–2.6)	0.89 (0.49–1.63)	0.95 (0.47–1.94)	1.05 (0.55–2)	0.9 (0.45–1.84)	1.73 (0.96–3.12)	1.74 (0.89–3.42)
Physical activity	*N* = 62 (19.9%)		*N* = 91 (29.2%)		*N* = 75 (24%)		*N* = 84 (26.9%)	
As before	**Ref.**	**Ref.**	**Ref.**	**Ref.**	**Ref.**	**Ref.**	**Ref.**	**Ref.**
Higher	0.52 (0.19–1.39)	0.51 (0.18–1.42)	**0.22 (0.06–0.74)**	**0.19 (0.05–0.73)**	2.8 (0.7–11.24)	2.32 (0.55–9.88)	**13.85 (1.68–114.09)**	**17.38 (2.06–146.3)**
Lower	1.89 (0.81–4.37)	1.8 (0.77–4.24)	**2.86 (1.33–6.16)**	**2.97 (1.33–6.61)**	**6.74 (1.94–23.41)**	**7.6 (2.1–27.55)**	**27.04 (3.61–202.73)**	**29.64 (3.9–225.14)**
Never	2.62 (0.67–10.24)	3.31 (0.76–14.34)	**3.62 (1.09–12.07)**	**4.3 (1.22–15.12)**	4.73 (1–22.38)	4.15 (0.85–20.38)	**25.5 (2.68–242.6)**	**31.47 (3.17–311.94)**
Freq. of PA	*N* = 62 (19.9%)		*N* = 91 (29.2%)		*N* = 75 (24%)		*N* = 84 (26.9%)	
30–60 min								
Never	**Ref.**	**Ref.**	**Ref.**	**Ref.**	**Ref.**	**Ref.**	**Ref.**	**Ref.**
Every day or almost	**0.33 (0.14–0.75)**	**0.27 (0.11–0.65)**	**0.21 (0.1–0.47)**	**0.2 (0.09–0.48)**	**0.19 (0.07–0.48)**	**0.17 (0.06–0.44)**	**0.39 (0.19–0.8)**	**0.44 (0.21–0.93)**
3–4 times a week	0.43 (0.17–1.08)	**0.37 (0.14–0.98)**	**0.33 (0.15–0.72)**	**0.34 (0.15–0.77)**	**0.34 (0.15–0.78)**	**0.29 (0.12–0.7)**	0.48 (0.22–1.07)	0.52 (0.23–1.16)
Once to twice a week	0.53 (0.2–1.4)	0.49 (0.18–1.34)	0.67 (0.29–1.52)	0.61 (0.25–1.49)	1.32 (0.55–3.15)	1.22 (0.5–2.98)	0.48 (0.19–1.19)	0.5 (0.19–1.3)
Once a week	0.45 (0.15–1.39)	0.36 (0.11–1.19)	1 (0.4–2.48)	1.08 (0.42–2.8)	0.69 (0.26–1.83)	0.78 (0.28–2.16)	0.54 (0.19–1.54)	0.53 (0.18–1.56)
> 60 min	*N* = 62 (19.9%)		*N* = 91 (29.2%)		*N* = 75 (24%)		*N* = 84 (26.9%)	
Never	**Ref.**	**Ref.**	**Ref.**	**Ref.**	**Ref.**	**Ref.**	**Ref.**	**Ref.**
Every day or almost	0.47 (0.15–1.45)	0.52 (0.16–1.66)	0.23 (0.05–1.06)	0.25 (0.05–1.2)	**0.1 (0.01–0.77)**	**0.09 (0.01–0.69)**	0.61 (0.24–1.54)	0.67 (0.26–1.75)
3–4 times a week	0.4 (0.11–1.44)	0.38 (0.1–1.39)	**0.13 (0.02–1)**	**0.12 (0.01–1.03)**	0.23 (0.05–1.05)	0.24 (0.05–1.16)	1.04 (0.35–3.07)	0.91 (0.29–2.8)
Once–twice a week	1.41 (0.6–3.34)	1.33 (0.55–3.22)	0.65 (0.28–1.54)	0.74 (0.31–1.78)	0.91 (0.35–2.35)	0.74 (0.27–2.04)	0.67 (0.24–1.84)	0.64 (0.23–1.79)
Once a week	0.86 (0.35–2.08)	0.85 (0.34–2.11)	0.76 (0.34–1.69)	0.77 (0.33–1.76)	0.78 (0.34–1.81)	0.72 (0.3–1.74)	0.49 (0.19–1.3)	0.49 (0.18–1.34)
Sleep quality	*N* = 62 (19.9%)		*N* = 91 (29.2%)		*N* = 75 (24%)		*N* = 84 (26.9%)	
As before	**Ref.**	**Ref.**	**Ref.**	**Ref.**	**Ref.**	**Ref.**	**Ref.**	**Ref.**
Worse	0.73 (0.38–1.39)	0.74 (0.38–1.45)	1.32 (0.76–2.31)	1.24 (0.69–2.22)	1.1 (0.57–2.12)	1.15 (0.59–2.27)	0.84 (0.47–1.51)	0.85 (0.46–1.56)
Better	**0.40 (0.15–1.02)**	**0.38 (0.15–1)**	1.17 (0.52–2.63)	1.27 (0.55–2.93)	1.7 (0.82–3.51)	1.63 (0.75–3.55)	0.79 (0.35–1.77)	0.78 (0.34–1.81)
Smoker	*N* = 62 (19.9%)			*N* = 91 (29.2%)		*N* = 75 (24%)		*N* = 84 (26.9%)
Non-smoker	**Ref.**	**Ref.**	**Ref.**	**Ref.**	**Ref.**	**Ref.**	**Ref.**	**Ref.**
Former smoker	**3.04 (1.11–8.28)**	**3.5 (1.23–9.95)**	0.83 (0.46–1.48)	0.86 (0.46–1.6)	1.08 (0.58–2.04)	1.04 (0.54–2)	1.01 (0.57–1.8)	1.08 (0.58–2.01)
Current smoker	1.63 (0.83–3.17)	1.78 (0.88–3.6)	1.3 (0.63–2.69)	1.38 (0.63–3.03)	1.12 (0.53–2.37)	1.08 (0.48–2.39)	0.66 (0.28–1.53)	0.77 (0.32–1.88)
Smoking	*N* = 62 (19.9%)		*N* = 91 (29.2%)		*N* = 75 (24%)		*N* = 84 (26.9%)	
As before	**Ref.**	**Ref.**	**Ref.**	**Ref.**	**Ref.**	**Ref.**	**Ref.**	**Ref.**
Higher	1.84 (0.43–7.77)	2.31 (0.52–10.31)	2 (0.5–8)	1.85 (0.43–7.97)	**5.95 (1.22–28.95)**	**5.66 (1.04–30.96)**	3.4 (0.62–18.75)	4.09 (0.67–24.97)
Lower	1.33 (0.45–3.95)	1.81 (0.57–5.73)	1.05 (0.36–3.08)	1.03 (0.33–3.2)	1.88 (0.63–5.59)	1.76 (0.57–5.39)	1.92 (0.66–5.58)	1.77 (0.59–5.32)
Never	0.51 (0.24–1.07)	**0.40 (0.18–0.89)**	1.19 (0.42–3.38)	1.11 (0.36–3.38)	1.73 (0.58–5.11)	1.68 (0.54––5.18)	1.98 (0.67–5.84)	1.72 (0.56–5.27)
Days of confinement	*N* = 62 (19.9%)		*N* = 91 (29.2%)		*N* = 75 (24%)		*N* = 84 (26.9%)	
> 50	**Ref.**	**Ref.**	**Ref.**	**Ref.**	**Ref.**	**Ref.**	**Ref.**	**Ref.**
45–50	1.08 (0.48–2.45)	1.08 (0.46–2.54)	0.50 (0.2–1.24)	0.51 (0.19–1.37)	0.67 (0.2–2.27)	0.57 (0.15–2.09)	0.74 (0.28–1.99)	0.75 (0.27–2.1)
40–45	0.67 (0.3–1.49)	0.67 (0.3–1.51)	0.78 (0.37–1.63)	0.83 (0.38–1.81)	1.49 (0.56–4.01)	1.35 (0.49–3.75)	1.02 (0.44–2.33)	0.99 (0.42–2.33)
< 40	0.60 (0.23–1.54)	0.64 (0.24–1.69)	0.61 (0.28–1.32)	0.54 (0.23–1.24)	1.29 (0.47–3.51)	1.26 (0.45–3.56)	0.87 (0.36–2.07)	0.77 (0.31–1.91)

^1^^a^Univariate regression model (model 1)

^b^Multivariate-adjusted model (model 2). Model 2 adjusted by: sex (men and women), educational level (postgraduate; university; high school; primary and other studies), housing during confinement (with or without children; with elderly; and children and elderly; residence or shared flat) and type of AP (high, medium, low)

^c^During the confinement, do you spend more time cooking?

^d^Time spent with family (eating together more often). ORs of the association between each studied variable related to eating and lifestyle habits and the weight gain change were mutually adjusted by each other. Statistically significant ORs are highlighted in bold. MedDiet: Mediterranean diet

The association between the risk of weight gain and PA levels was also examined. The results showed that those individuals who reduced their PA levels during confinement or were sedentary increased the risk of weight gain by fourfold. In addition, those who were active 30–60 min every day or 3–4 times a week reduced the weight gain risk by 73% and 60%, respectively. This trend was also observed for all age groups in participants that spent more than 60 min doing PA.

Better sleep quality was also associated with a lower risk of weight gain (OR = 0.38, CI 0.15–1, *p* < 0.05). In addition, significant associations were found between sleep time and weight gain.

Finally, for smoking habits, the youngest former smokers had a higher risk of weight gain (OR = 3.5, CI 1.23–9.95, *p* = 0.019). In comparison to those whose smoking patterns were not modified, participants that never smoked or reduced their pattern of consumption showed a significantly lower risk of weight gain (almost 50%).

### Associations between the Mediterranean pattern and weight gain during lockdown

**Fig. 1:**
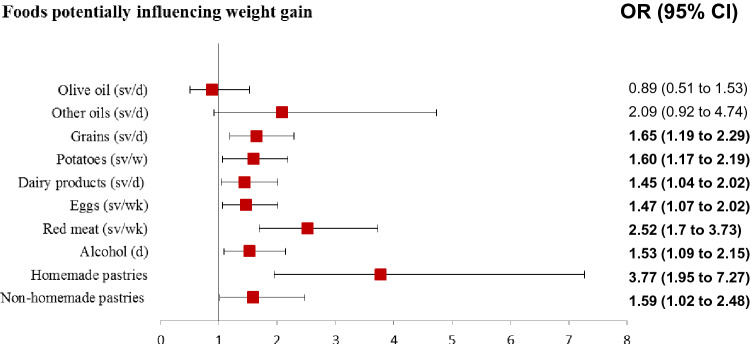
Foods potentially influencing weight gain. ORs of the association between each studied variable related to eating and lifestyle habits and weight gain change were mutually adjusted by each other. Bold: statistically significant ORs (*P* < 0.05)

As detailed in Fig. 1, Table 5 and Supplementary Table 3, an increase in the consumption of olive oil or other types of fats was not linked to a higher risk of weight gain. However, maintaining its consumption was associated with a lower risk of weight gain by 54%. Although it was not statistically significant, an increase in the consumption of other types of fats was associated with a higher risk (almost twofold more, *p* = 0.077). Increased consumption of fresh fruit (≥3 serving/day) was also associated with a lower risk of weight gain. On the other hand, increasing the intake of cereals, potatoes, dairy products, and eggs was directly associated with a higher risk of weight gain (OR = 1.65, CI 1.19–2.29, *p* = 0.003; OR = 1.60, CI 1.17–2.19, *p* = 0.003; OR = 1.44, CI 1.04–2.02, *p* = 0.030; OR = 1.47, CI 1.07–2.02, *p* = 0.016, respectively).

**Table 5:** Associations between MedDiet pattern and weight gain stratified by age in regarding to the COVID-19 confinement

	≤ 33 y		34–44 y		44–53 y		≥ 53 y	
	Crude model^a^	Adjusted model^b^	Crude model^a^	Adjusted model^b^	Crude model^a^	Adjusted model^b^	Crude model^a^	Adjusted model^b^
	OR (95% CI)	OR (95% CI)	OR (95% CI)	OR (95% CI)	OR (95% CI)	OR (95% CI)	OR (95% CI)	OR (95% CI)
Olive oil (sv/d)	*N* = 62 (19.9%)		*N* = 91 (29.2%)		*N* = 75 (24%)		*N* = 84 (26.9%)	
Unknown	**Ref.**	**Ref.**	**Ref.**	**Ref.**	**Ref.**	**Ref.**	**Ref.**	**Ref.**
No	**0.34 (0.15–0.77)**	**0.31 (0.12–0.76)**	**0.30 (0.1–0.95)**	0.37 (0.1–1.45)	0.65 (0.21–1.94)	0.67 (0.2–2.22)	1.04 (0.3–3.54)	1.21 (0.33–4.48)
Yes	0.64 (0.26–1.53)	0.69 (0.26–1.85)	**0.30 (0.09–0.98)**	0.35 (0.09–1.42)	1.07 (0.35–3.3)	1.04 (0.3–3.56)	1.85 (0.52–6.56)	2.61 (0.66–10.28)
Other oils (sv/d)	*N* = 62 (19.9%)		*N* = 91 (29.2%)		*N* = 75 (24%)		*N* = 84 (26.9%)	
Unknown	**Ref.**	**Ref.**	**Ref.**	**Ref.**	**Ref.**	**Ref.**	**Ref.**	**Ref.**
No	**0.27 (0.11–0.69)**	**0.21 (0.07–0.61)**	0 (0–0)	0 (0–0)	0.47 (0.11–1.94)	0.68 (0.15–3.04)	0.63 (0.17–2.43)	0.57 (0.12–2.74)
Yes	0.93 (0.29–3.01)	0.61 (0.16–2.3)	0 (0–0)	0 (0–0)	4 (0.5–31.98)	6.74 (0.67–67.7)	2.92 (0.44–19.23)	3.53 (0.37–33.17)
Nuts (sv/d)	*N* = 62 (19.9%)		*N* = 91 (29.2%)		*N* = 75 (24%)		*N* = 84 (26.9%)	
No	**Ref.**	**Ref.**	**Ref.**	**Ref.**	**Ref.**	**Ref.**	**Ref.**	**Ref.**
Yes	1.14 (0.62–2.09)	1.23 (0.64–2.38)	1.2 (0.69–2.08)	0.98 (0.53–1.84)	**1.82 (1–3.32)**	**1.97 (1.01–3.85)**	1.44 (0.80–2.62)	1.62 (0.83–3.14)
Nuts (sv/d)	*N* = 62 (19.9%)		*N* = 91 (29.2%)		*N* = 75 (24%)		*N* = 84 (26.9%)	
0	**Ref.**	**Ref.**	**Ref.**	**Ref.**	**Ref.**	**Ref.**	**Ref.**	**Ref.**
1–2	1.31 (0.52–3.35)	1.31 (0.48–3.63)	0.73 (0.31–1.73)	0.55 (0.19–1.61)	1.77 (0.59–5.33)	1.62 (0.5–5.31)	1.2 (0.46–3.09)	0.9 (0.31–2.67)
2–3	1.35 (0.51–3.53)	1.69 (0.58–4.92)	0.8 (0.33–1.95)	0.57 (0.19–1.71)	1.33 (0.41–4.25)	1.39 (0.39–4.96)	1.35 (0.5–3.69)	1.02 (0.33–3.18)
> 3	1.37 (0.53–3.56)	1.81 (0.62–5.25)	0.56 (0.23–1.34)	**0.34 (0.11–1.02)**	1.45 (0.44–4.73)	1.76 (0.48–6.47)	1.09 (0.41–2.87)	0.87 (0.29–2.61)
Vegetables (sv/d)	*N* = 62 (19.9%)		*N* = 91 (29.2%)		*N* = 75 (24%)		*N* = 84 (26.9%)	
No	**Ref.**	**Ref.**	**Ref.**	**Ref.**	**Ref.**	**Ref.**	**Ref.**	**Ref.**
Yes	0.89 (0.47–1.67)	1.02 (0.51–2.03)	0.7 (0.39–1.24)	0.59 (0.3–1.16)	0.75 (0.41–1.36)	0.73 (0.38–1.39)	0.79 (0.44–1.44)	0.85 (0.44–1.65)
Vegetables (sv/d)	*N* = 62 (19.9%)		*N* = 91 (29.2%)		*N* = 75 (24%)		*N* = 84 (26.9%)	
0	**Ref.**	**Ref.**	**Ref.**	**Ref.**	**Ref.**	**Ref.**	**Ref.**	**Ref.**
1–2	1.19 (0.23–6.15)	1.99 (0.29–13.44)	0 (0–0)	0 (0–0)	0.48 (0.12–1.88)	0.43 (0.1–1.89)	0 (0–0)	0 (0–0)
≥ 2	0.76 (0.14–4.05)	1.21 (0.17–8.45)	0 (0–0)	0 (0–0)	0.27 (0.06–1.12)	**0.20 (0.04–0.94)**	0 (0–0)	0 (0–0)
*Sofrito* (sv/d)	*N* = 62 (19.9%)		*N* = 91 (29.2%)		*N* = 75 (24%)		*N* = 84 (26.9%)	
No	**Ref.**	**Ref.**	**Ref.**	**Ref.**	**Ref.**	**Ref.**	**Ref.**	**Ref.**
Yes	1.11 (0.6–2.06)	1.21 (0.62–2.4)	1.19 (0.7–2.05)	1.45 (0.76–2.77)	1.5 (0.81–2.77)	1.3 (0.67–2.53)	1.01 (0.54–1.92)	1.14 (0.56–2.31)
Fresh fruit (sv/d)	*N* = 62 (19.9%)		*N* = 91 (29.2%)		*N* = 75 (24%)		*N* = 84 (26.9%)	
No	**Ref.**	**Ref.**	**Ref.**	**Ref.**	**Ref.**	**Ref.**	**Ref.**	**Ref.**
Yes	1.09 (0.58–2.05)	1.09 (0.54–2.19)	1.21 (0.7–2.08)	1.07 (0.57–2.01)	1.04 (0.56–1.92)	0.92 (0.47–1.81)	1.24 (0.68–2.27)	1.17 (0.6–2.28)
Fresh fruit (sv/d)	*N* = 62 (19.9%)		*N* = 91 (29.2%)		*N* = 75 (24%)		*N* = 84 (26.9%)	
0	**Ref.**	**Ref.**	**Ref.**	**Ref.**	**Ref.**	**Ref.**	**Ref.**	**Ref.**
1–2	0.5 (0.21–1.22)	0.5 (0.19–1.33)	0.53 (0.2–1.42)	0.31 (0.08–1.18)	0.45 (0.13–1.55)	0.23 (0.05–1)	1.02 (0.27–3.78)	1.4 (0.35–5.5)
2–3	0.67 (0.26–1.7)	0.96 (0.34–2.72)	0.65 (0.22–1.9)	0.39 (0.09–1.64)	0.37 (0.1–1.39)	**0.24 (0.05–1.1)**	0.53 (0.13–2.11)	0.71 (0.17–3.01)
≥ 3	**0.11 (0.01–0.99)**	0.13 (0.01–1.18)	0.55 (0.14–2.12)	0.31 (0.05–1.75)	0.42 (0.08–2.25)	**0.26 (0.04–1.65)**	0.71 (0.15–3.22)	1.03 (0.2–5.17)
Legumes (sv/wk)	*N* = 62 (19.9%)		*N* = 91 (29.2%)		*N* = 75 (24%)		*N* = 84 (26.9%)	
No	**Ref.**	**Ref.**	**Ref.**	**Ref.**	**Ref.**	**Ref.**	**Ref.**	**Ref.**
Yes	1.19 (0.62–2.3)	1.20 (0.59–2.44)	0.88 (0.49–1.58)	0.71 (0.36–1.4)	1.28 (0.67–2.45)	1.63 (0.79–3.37)	1.16 (0.6–2.24)	1.20 (0.58–2.47)
Grains (sv/d)	*N* = 62 (19.9%)		*N* = 91 (29.2%)		*N* = 75 (24%)		*N* = 84 (26.9%)	
No	**Ref.**	**Ref.**	**Ref.**	**Ref.**	**Ref.**	**Ref.**	**Ref.**	**Ref.**
Yes	1.77 (0.96–3.29)	1.86 (0.95–3.64)	1.63 (0.93–2.86)	1.74 (0.91–3.3)	1.79 (0.92–3.48)	1.45 (0.7–3.01)	1.47 (0.72–3)	1.20 (0.55–2.62)
Whole grains (sv/d)	*N* = 62 (19.9%)		*N* = 91 (29.2%)		*N* = 75 (24%)		*N* = 84 (26.9%)	
No	**Ref.**	**Ref.**	**Ref.**	**Ref.**	**Ref.**	**Ref.**	**Ref.**	**Ref.**
Yes	0.84 (0.41–1.68)	0.98 (0.45–2.12)	0.79 (0.34–1.83)	0.65 (0.26–1.63)	1.74 (0.64–4.71)	1.29 (0.44–3.77)	1.64 (0.72–3.74)	1.44 (0.59–3.52)
Potatoes (sv/wk)	*N* = 62 (19.9%)		*N* = 91 (29.2%)		*N* = 75 (24%)		*N* = 84 (26.9%)	
No	**Ref.**	**Ref.**	**Ref.**	**Ref.**	**Ref.**	**Ref.**	**Ref.**	**Ref.**
Yes	1.58 (0.86–2.92)	1.54 (0.8–2.98)	1.50 (0.88–2.55)	**1.94 (1.04–3.64)**	1.48 (0.81–2.71)	1.43 (0.74–2.78)	1.62 (0.84–3.09)	1.61 (0.79–3.27)
Dairy products (sv/d)	*N* = 62 (19.9%)		*N* = 91 (29.2%)		*N* = 75 (24%)		*N* = 84 (26.9%)	
No	**Ref.**	**Ref.**	**Ref.**	**Ref.**	**Ref.**	**Ref.**	**Ref.**	**Ref.**
Yes	**1.92 (1.05–3.54)**	**2.21 (1.11–4.39)**	1.2 (0.67–2.15)	1.29 (0.65–2.53)	1.39 (0.72–2.68)	1.51 (0.74–3.08)	1.25 (0.6–2.6)	1.14 (0.5–2.63)
Eggs (sv./wk)	*N* = 62 (19.9%)		*N* = 91 (29.2%)		*N* = 75 (24%)		*N* = 84 (26.9%)	
No	**Ref.**	**Ref.**	**Ref.**	**Ref.**	**Ref.**	**Ref.**	**Ref.**	**Ref.**
Yes	1.7 (0.91–3.17)	**1.97 (1–3.86)**	1.64 (0.95–2.84)	1.45 (0.77–2.71)	1.23 (0.64–2.34)	0.92 (0.45–1.89)	1.36 (0.73–2.52)	1.35 (0.68–2.64)
Red meat (sv/wk)	*N* = 62 (19.9%)		*N* = 91 (29.2%)		*N* = 75 (24%)		*N* = 84 (26.9%)	
No	**Ref.**	**Ref.**	**Ref.**	**Ref.**	**Ref.**	**Ref.**	**Ref.**	**Ref.**
Yes	**2.44 (1.23–4.86)**	**2.53 (1.18–5.41)**	1.90 (0.95–3.82)	1.91 (0.85–4.3)	**3.09 (1.42–6.72)**	**3.37 (1.42–8.02)**	**3.45 (1.44–8.29)**	**2.98 (1.14–7.76)**
Lean meat (sv/wk)	*N* = 62 (19.9%)		*N* = 91 (29.2%)		*N* = 75 (24%)		*N* = 84 (26.9%)	
No	**Ref.**	**Ref.**	**Ref.**	**Ref.**	**Ref.**	**Ref.**	**Ref.**	**Ref.**
Yes	1.45 (0.64–3.26)	1.57 (0.64–3.83)	0.84 (0.39–1.82)	0.95 (0.38–2.37)	1.71 (0.75–3.92)	1.51 (0.6–3.83)	1.61 (0.69–3.78)	1.64 (0.62–4.34)
Fish and seafood (sv/w) (srv/wk)	*N* = 62 (19.9%)		*N* = 91 (29.2%)		*N* = 75 (24%)		*N* = 84 (26.9%)	
No	**Ref.**	**Ref.**	**Ref.**	**Ref.**	**Ref.**	**Ref.**	**Ref.**	**Ref.**
Yes	1.69 (0.88–3.23)	**2.05 (1.01–4.19)**	1.02 (0.52–2.01)	1.2 (0.54–2.65)	1.46 (0.69–3.11)	1.82 (0.78–4.22)	0.73 (0.34–1.56)	0.66 (0.29–1.49)
Alcohol (d)	*N* = 62 (19.9%)		*N* = 91 (29.2%)		*N* = 75 (24%)		*N* = 84 (26.9%)	
No	**Ref.**	**Ref.**	**Ref.**	**Ref.**	**Ref.**	**Ref.**	**Ref.**	**Ref.**
Yes	1.81 (0.81–4.03)	1.56 (0.66–3.71)	0.78 (0.44–1.41)	1.01 (0.51–1.99)	**1.93 (1.05–3.55)**	**2.49 (1.24–5.03)**	**2.01 (1.05–3.85)**	**2.02 (0.98–4.18)**
Sweetened beverages (d)	*N* = 62 (19.9%)		*N* = 91 (29.2%)		*N* = 75 (24%)		*N* = 84 (26.9%)	
No	**Ref.**	**Ref.**	**Ref.**	**Ref.**	**Ref.**	**Ref.**	**Ref.**	**Ref.**
Yes	1.46 (0.57–3.78)	1.41 (0.49–4.06)	1.86 (0.86–4)	1.25 (0.53–2.96)	1.18 (0.37–3.74)	1.29 (0.36–4.64)	1.59 (0.52–4.91)	1.74 (0.48–6.25)
Juices (≥ 1 /d)	*N* = 62 (19.9%)		*N* = 91 (29.2%)		*N* = 75 (24%)		*N* = 84 (26.9%)	
No	**Ref.**	**Ref.**	**Ref.**	**Ref.**	**Ref.**	**Ref.**	**Ref.**	**Ref.**
Yes	0.98 (0.51–1.89)	0.99 (0.49–2.02)	1.13 (0.64–1.99)	1.27 (0.66–2.43)	1.16 (0.62–2.15)	0.99 (0.5–1.99)	0.92 (0.51–1.66)	1.1 (0.57–2.13)
Homemade pastries	*N* = 62 (19.9%)		*N* = 91 (29.2%)		*N* = 75 (24%)		*N* = 84 (26.9%)	
As before	**Ref.**	**Ref.**	**Ref.**	**Ref.**	**Ref.**	**Ref.**	**Ref.**	**Ref.**
No	0.43 (0.14–1.38)	0.36 (0.11–1.25)	1.14 (0.44–2.95)	1.41 (0.48–4.1)	0.87 (0.34–2.23)	0.99 (0.35–2.79)	1.02 (0.48–2.19)	1.07 (0.46–2.49)
Yes	1.34 (0.53–3.35)	1.07 (0.4–2.88)	1.92 (0.8–4.63)	2.01 (0.75–5.39)	1.12 (0.46–2.75)	1.35 (0.51–3.57)	1.89 (0.89–4)	**2.31 (0.99–5.39)**
Non-homemade pastries	*N* = 62 (19.9%)		*N* = 91 (29.2%)		*N* = 75 (24%)		*N* = 84 (26.9%)	
As before	**Ref.**	**Ref.**	**Ref.**	**Ref.**	**Ref.**	**Ref.**	**Ref.**	**Ref.**
No	1.01 (0.32–3.2)	1.01 (0.29–3.5)	0.93 (0.37–2.32)	1.14 (0.4–3.28)	**1.23 (0.42–3.58)**	**1.41 (0.44–4.5)**	**0.89 (0.36–2.2)**	**0.93 (0.34–2.56)**
Yes	3.53 (0.96–12.91)	2.91 (0.73–11.65)	2.04 (0.64–6.52)	2.81 (0.73–10.76)	**14.93 (3.01–74.04)**	**13.74 (2.49–75.83)**	**4.38 (1.21–15.81)**	**4.62 (1.05–20.25)**

^a^Univariate regression model (model 1)

^b^Multivariate-adjusted model (model 2). Model 2 adjusted by: sex (men and women), educational level (postgraduate; university; high school; primary and other studies), housing during confinement (with or without children; with elderly; and children and elderly; residence or shared flat) and type of AP (high, medium, low). ORs of the association between each studied variable related to eating and lifestyle habits and weight gain change were mutually adjusted by each other. Statistically significant ORs are highlighted in bold. *d* day, *sv* serving, *wk* week

Regarding meat consumption, significant associations were observed between a higher consumption of red meat and the risk of weight gain for all ages (between 2.5- and 3.4-fold more).

In the case of pastries (including homemade and non-homemade), their intake was strongly associated with a higher risk of weight gain. Non-homemade pastries showed a higher risk compared to homemade pastries (377% vs. 59%).

Finally, an increase in alcohol consumption was linked to a higher risk of increasing weight (by 53%). Participants aged between 44 and 52 years and those aged ≥53 years showed a higher risk (OR = 2.49, CI 1.24–5.03, *p* = 0.011; OR = 2.02, CI 0.98–4.18, *p* = 0.050, respectively). In addition, an increase in daily sweetened beverage intake was associated with a higher risk of weight gain by 55%, although this was not significant (*p* = 0.070). Drinking more than eight glasses of water a day also reduced the risk (OR = 0.65, CI 0.42–1.0, *p* < 0.05; data not shown).

### MedDiet score and lifestyle

After analysing the MedDiet adherence scores and the lifestyles of the participants, 27 different patterns were identified. These patterns resulted from a combination of changes in diet [improvement (+), no changes (±) and a decrease of dietary quality (−)], PA levels [improvement (+), no changes (0) and decrease of PA (−)] and sleep quality [improvement (+), no changes (=) and decrease of quality sleep (−)] (Table 6). In general, those participants who showed a high adherence to the MedDiet had a lower risk of weight gain in comparison to those who showed low-to-medium adherence. Moreover, the results show that PA plays an important role in body weight maintenance. A medium or low adherence together with low or null PA levels, or even a reduced PA during lockdown, was associated with a higher risk of weight gain. In relation to quality of sleep, it was also observed that a maintained or worse sleep was associated with a higher risk of weight gain. In conclusion, the highest risk was observed in participants with a low MedDiet adherence, low or null PA, and a poorer quality of sleep.

**Table 6:** Patterns identified according to Mediterranean diet Score and lifestyle (PA and quality of sleep) during the COVID-19 confinement

Adherence	Physical activity	Quality of sleep	Higher risk of weight gain	*P* value
				< 0.001
(+)	(+)	(+)	5 (21.7)	
(+)	(+)	(−)	6 (27.3)	
(+)	(+)	(=)	5 (12.5)	
(+)	(−)	(+)	7 (33.3)	
(+)	(−)	(−)	23 (33.8)	
(+)	(−)	( =)	37 (37.4)	
(+)	0	( +)	0 (0)	
(+)	0	(–)	2 (8.7)	
(+)	0	(=)	6 (15.8)	
(±)	(+)	(+)	1 (11.1)	
(±)	(+)	(−)	3 (18.8)	
(±)	(+)	(=)	2 (6.7)	
(±)	(−)	(+)	10 (50)	
(±)	(−)	(−)	12 (35.3)	
(±)	(−)	(=)	22 (45.8)	
(±)	0	(+)	3 (50)	
(±)	0	(−)	5 (41.7)	
(±)	0	(=)	8 (34.8)	
(−)	(+)	(+)	4 (30.8)	
(−)	(+)	(−)	9 (33.3)	
(−)	(+)	(=)	7 (15.6)	
(−)	(−)	(+)	4 (25)	
(−)	(−)	(−)	5 (22.7)	
(−)	(−)	(=)	5 (17.2)	
(−)	0	(+)	12 (42.9)	
(−)	0	(−)	40 (46.5)	
(−)	0	(=)	51 (53.7)	

^*^Values are *n* (%). ¥*χ*^2^-test for categorical variable. (+): high adherence or PA and better quality of sleep; (−): low adherence to MedDiet or PA, 0: include low or null PA; (±): medium adherence; = quality of sleep maintained

## Discussion

This study provides information about the changes in dietary habits and lifestyle among the Spanish population during the COVID-19 lockdown. The data collected by an online survey between April 23 and June 2, 2020 suggest that the population studied adopted healthier dietary habits, which was associated with a higher MedDiet adherence. This improvement was mainly observed in the older group (≥53 years old), who reported a higher adherence to the MedDiet than the youngest group. Nevertheless, this study also revealed that the COVID-19 lockdown had a negative effect on PA and sleep quality, although these changes were to a relatively lower degree.

### Adherence to a MedDiet and lifestyle adaptations during the COVID-19 lockdown

Higher adherence to a MedDiet was observed in the older Spanish population (mean of MEDAS score 9.4 ± 1.6) as compared to the rest of the participants (mean of MEDAS score 8.7 ± 1.6) during lockdown. These results are aligned with the findings reported by Rodríguez-Pérez et al. [20], which showed that Spanish adults aged 51 years and older have the highest MedDiet adherence. León-Muñoz et al. [21], in agreement with our results, also found that Spanish adults aged ≥45 years show a higher adherence to the MedDiet. It should be noted that the short MEDAS screener used in the current study has only been validated in older Spanish adults. However, as this tool has been widely used in a large number of studies among individuals with different ages, and has proven reliable when assessing different populations (European, German, Spanish, English, etc.), the MEDAS may be used as a simple, reliable, and effective tool to measure adherence to the MedDiet [18, 19, 22, 23].

One possible explanation for the improvement in dietary habits during the state of emergency is that people likely had more time to cook and organise their meals at home. In recent decades, the population of the USA and Europe have reduced the time spent cooking and preparing meals due to the stress of daily life and a lack of free time [24]. The current results show that most of the study population followed a healthy eating pattern during lockdown. The majority of the participants did not increase food consumption or snacks between meals, and did not skip meals. In addition, the participants reported having between 3 and 4 meals a day, choosing both fresh, frozen, and locally produced products, limiting the consumption of fast or convenience food, and maintaining a moderate intake of fried foods during home confinement. Interestingly, the survey also revealed that the participants increased the time that they spent cooking and preferred healthier types of cooking techniques, such as roasting, using a griddle, boiling or steaming, and consuming casseroles or stews.

Most of the participants also considered the quality of their meals to be better as compared to the period before the lockdown. In contrast to the these results, a previous study [10] reported that the foods purchased by Spanish households during the pandemic became worse in quality (higher energy density and lower nutritional quality) and resulted in an unhealthier dietary pattern compared to the pre-COVID-19 period. However, the authors reported several limitations of this study, such as following the dietary patterns of certain vulnerable groups (e.g. migrants and refugees) that may be very different from the rest of the Spanish population. In addition, economic aspects (e.g. income levels) that may influence dietary patterns were not considered.

### Body weight management

According to several studies, dietary quality is a more important factor for weight loss than dietary quantity [24, 25]. In the current study, 33% of the participants reported weight gain, 12.4% maintained their body weight, and 46% declared a weight loss. Most of the participants that reported an increase or a reduction in the quantity of foods consumed, also reported improvements in the quality of their diets. This might explain why the preference choice for snacking between meals was fresh fruits and vegetables, followed by chocolate and natural nuts. These data are similar to that reported by Laguna et al. [12], which showed that the Spanish population studied (362 individuals) preferred pasta and vegetables to achieve a healthy status, and nuts, cheese, and chocolate to improve their mood. These authors also reported that, to maintain their body weight, the participants reduced purchases of unhealthy foods, such as sugary baked goods and desserts. We also observed that participants who ate more and a lower quality of food, or those who ate more and a better quality of food, were at a higher risk of increasing their body weight. Eating more, especially foods with a low nutritional quality, was probably driven by the anxiety and boredom caused by the COVID-19 lockdown [6]. Other studies have shown that, under these non-standard living conditions, there are increases in eating and the overconsumption of superfluous food rich in sugar, fats and salt [25]. The consumption of junk foods are directly associated with a higher risk of developing obesity and metabolic disorders, and increase the risk of complications from COVID-19 [13, 26].

In addition, participants that reported an increase in their consumption of refined cereals, potatoes, dairy products, eggs, red meat, and alcohol showed a higher risk of weight gain. These results are in agreement with other studies that have shown that the consumption of higher quantities of red and processed meats and refined cereals is a risk factor for obesity [27]. Currently, there is controversy about whether the consumption of eggs is associated with an increased risk of weight gain [28]. In relation to dairy products, the present evidence suggests that consumption of these products does not cause weight gain, and can increase lean body mass and reduce body fat; however, firmly establishing these associations will require further research [29, 30]. A higher risk of weight gain with dairy products is likely associated with an increase in the consumption of sugary and fruit yogurts or cured cheese. In relation to potatoes, there is not any conclusive evidence linking potato intake with the risk of developing obesity; however, some authors have reported a positive association between obesity and the intake of French fries [29].

Similar to other studies [30, 31], the current data show body weight gain was not observed in individuals who increased olive oil consumption. In contrast, the consumption of other types of oils and fats were directly associated with body weight gain, as reported by other studies [32, 33].

### Other lifestyle habits

Although the study population had access to different home training programs (videos) and new technologies (social networking and apps) that promote PA [38], the results showed that individuals did not adequately maintain their PA levels during lockdown. These results are similar to those found in other studies [20, 34]. Restrictions on outdoor activities and reduced access to swimming pools and gyms have reduced overall PA time and promoted both sedentary habits and obesity [35]. It is also possible that boredom, living with children and taking care of their education, prolonged screen time associated remote working, and excessive time spent watching TV have also promoted sedentary behaviours [36, 37]. It is well known that one of the major risk factors for chronic diseases associated with the immune system is ageing, but evidence suggests that regular PA, along with healthy dietary habits and a healthy lifestyle, can help reduce the incidence of these diseases [38].

The COVID-19 pandemic is also associated with increased sadness, fear, and anxiety, which can reduce sleep quality [34, 39]. In the current study, a third of the participants reported a deterioration in sleep quality, while ~50% reported no changes compared to the period before the lockdown. Previous studies on the impacts of social isolation on both psychological well-being and sleep quality have highlighted the role of the following factors: decreased sunlight exposure, dietary changes, environmental temperature due to confinement, reduced social interaction, working longer hours under stressful circumstances, and living with uncertainty and insecurity about the state of health [40, 41]. The current results showed a significant association between sleep quality and weight gain in the youngest participants. Other studies have also reported that inadequate sleep increases the risk of weight gain during self-quarantine [42]. Indeed, decreasing sleep duration promotes obesity by increasing the risk of eating more [43]. There is the possibility that the people who reported weight gain slept worse, were awake longer, and thus had more opportunities to eat during the night hours.

There are several limitations to the current study. First, the online survey was self-reported, which have could led to inaccurate answers. We decided to use an online platform because collecting the data in this fashion was feasible, quick, and relatively easy for the participants. Online data collection is also highly recommended in the current pandemic situation [44]. In addition, a sampling bias can be associated with collecting information using online questionnaires. For example, the snowball sampling method can lead to an increased number of participants from a particular demographic category. In our case, most of the participants were Spanish (75% of the sample). Therefore, the current results can only be extrapolated to a similar population with a high educational level that lives mainly in urban areas. The results could also have been affected by other factors such as limited access to the Internet, the participants’ interest in health topics, available space to do PA, and personal factors (e.g. living in a rural or urban setting, job status, relationship status, and living arrangements). Finally, this was a cross-sectional study.

This study also has several strengths that should be mentioned. The survey covered all ages (>18 years) and several European territories. To our knowledge, this is the first study that has examined and compared changes associated with dietary habits and lifestyle in the Spanish adult population.

## Conclusion

From this study, it can be concluded that the Spanish population has improved their dietary habits during home lockdown. However, the COVID-19 pandemic has promoted certain unhealthy behaviours, such as increased snacking between meals, increased food intake, and an increase in sedentary behaviour, all possibly caused by anxiety and boredom. On the other hand, the pandemic allowed people to spend more time cooking, to share more meals with family members, and to choose healthier foods to improve dietary quality.

It is known that an inadequate intake of Mediterranean foods, together with an unhealthy lifestyle, can promote obesity, metabolic disorders, CVD, and cancer. Thus, knowing the negative impact of COVID-19 on health, it is necessary to design effective lifestyle interventions based on healthy diets, such as the MedDiet, during the ongoing pandemic. Interventions designed to improve dietary quality and other lifestyle behaviours, such as sleep quality and PA, could be the beginning of a long road to leading to the prevention of chronic diseases and complications associated with COVID-19.

## Supplementary Information

Below is the link to the electronic supplementary material.

Supplementary file1 (DOCX 117 KB)

Supplementary file2 (PDF 92 KB)

## Declarations

### Data available on request due to restrictions

The data presented in this study are available on request from the corresponding author. The data are not publicly available due to ethical concerns.
