# Supplementary material for: Changes in Spanish lifestyle and dietary habits during the COVID-19 lockdown
Source: Eur J Nutr. 2022 Feb 7;61(5):2417–34. doi: 10.1007/s00394-022-02814-1 (PMC9279186; doi:10.1007/s00394-022-02814-1)
Supplement: Supplementary file 1 — Supplementary file1 (DOCX 117 KB) [file 394_2022_2814_MOESM1_ESM.docx]

**Supplementary Table 1.** **Dietary and lifestyle adaptations during the COVID-19 lockdown**

|  |  |  | **< 33y. (Q1)** | **33-44y. (Q2)** | **44-53y. (Q3)** | **>53y. (Q4)** | **P-values were corrected for multiple comparisons γ** | | | | | | |
| --- | --- | --- | --- | --- | --- | --- | --- | --- | --- | --- | --- | --- | --- |
|  |  | **Spanish participants**  **N=945** | **N** | **N** | **N** | **N** | **P-value¥** | **Q1 vs Q2** | **Q2 vs Q3** | **Q3 vs Q4** | **Q1 vs Q3** | **Q2 vs Q4** | **Q1 vs Q4** |
| **Weight** | |  |  |  |  |  | 0.041 | 0.185 | 0.488 | 0.853 | 0.070 | 0.856 | 0.123 |
|  | As before | 436 (46.1) * | 114 (46.9) | 105 (42.3) | 107 (49.1) | 110 (46.6) | 0.516 |  |  |  |  |  |  |
|  | Unknown | 80 (8.5) | 29 (11.9) | 24 (9.7) | 11 (5) | 16 (6.8) | 0.038 | 0.445 | 0.158 | 0.281 | 0.035 | 0.409 | 0.172 |
|  | Higher | 312 (33.0) | 62 (25.5) | 91 (36.7) | 75 (34.4) | 84 (35.6) | 0.035 | 0.030 | 0.710 | 0.680 | 0.093 | 0.438 | 0.054 |
|  | Lower | 117 (12.4) | 38 (15.6) | 28 (11.3) | 25 (11.5) | 26 (11) | 0.359 |  |  |  |  |  |  |
| **Eating More** | |  |  |  |  |  | 0.100 |  |  |  |  |  |  |
|  | No | 398 (42.1) | 85 (35) | 102 (41.1) | 98 (45) | 113 (47.9) | 0.001 | 0.279 | 0.203 | 0.145 | 0.111 | 0.005 | <0.001 |
|  | As before | 221 (23.4) | 69 (28.4) | 54 (21.8) | 48 (22) | 50 (21.2) | 0.028 | 0.562 | 0.297 | 0.349 | 0.353 | 0.039 | 0.041 |
|  | Yes | 326 (34.5) | 89 (36.6) | 92 (37.1) | 72 (33) | 73 (30.9) | 0.025 | 0.285 | 0.425 | 0.254 | 0.317 | 0.068 | 0.012 |
| **Snacking Frequency** | |  |  |  |  |  | 0.001 | 0.791 | 0.357 | 0.359 | 0.268 | 0.005 | <0.001 |
|  | No | 527 (55.8) | 118 (48.6) | 128 (51.6) | 126 (57.8) | 155 (65.7) |  |  |  |  |  |  |  |
|  | Yes | 387 (41.0) | 113 (46.5) | 108 (43.5) | 87 (39.9) | 79 (33.5) |  |  |  |  |  |  |  |
| **Type of Snack** | |  |  |  |  |  |  |  |  |  |  |  |  |
|  | Chocolate | 406 (43.0) | 126 (20.8) | 116 (20.4) | 90 (22.7) | 74 (20.2) | <0.001 | 0.150 | 0.254 | 0.053 | 0.059 | 0.005 | <0.001 |
|  | Fried Foods | 51 (5.4) | 16 (2.6) | 20 (3.5) | 9 (2.3) | 6 (1.6) | 0.035 | 0.325 | 0.213 | 0.434 | 0.428 | 0.035 | 0.132 |
|  | Fried nuts | 52 (5.5) | 16 (2.6) | 21 (3.7) | 7 (1.8) | 8 (2.2) | 0.032 | 0.464 | 0.076 | 0.563 | 0.262 | 0.068 | 0.224 |
|  | Row nuts | 287 (30.4) | 88 (14.5) | 84 (14.8) | 58 (14.6) | 57 (15.6) | 0.011 | 0.327 | 0.156 | 0.525 | 0.066 | 0.059 | 0.018 |
|  | Salted snack | 116 (12.3) | 36 (6.0) | 44 (7.7) | 22 (5.5) | 14 (3.8) | <0.001 | 0.225 | 0.047 | 0.198 | 0.157 | <0.001 | 0.005 |
|  | Fruit & Vegetables | 387 (41.0) | 131 (21.7) | 108 (19) | 75 (18.9) | 73 (19.9) | <0.001 | 0.041 | 0.053 | 0.246 | <0.001 | 0.012 | 0.005 |
|  | Popcorn | 76 (8.0) | 25 (4.1) | 24 (4.2) | 14 (3.5) | 13 (3.6) | 0.146 |  |  |  |  |  |  |
|  | Chips | 188 (19.9) | 49 (8.1) | 60 (10.6) | 44 (11.1) | 35 (9.6) | 0.082 | 0.422 | 0.323 | 0.296 | 0.544 | 0.035 | 0.334 |
|  | Cereal pancake | 75 (7.9) | 35 (5.8) | 22 (3.9) | 10 (2.5) | 8 (2.2) | <0.001 | 0.110 | 0.096 | 0.340 | <0.001 | 0.039 | 0.005 |
|  | Pastries | 128 (13.5) | 51 (8.4) | 36 (6.3) | 24 (6) | 17 (4.6) | <0.001 | 0.112 | 0.161 | 0.201 | 0.015 | 0.028 | <0.001 |
|  | No snacking | 170 (18.0) | 32 (5.3) | 33 (5.8) | 44 (11.1) | 61 (16.7) | <0.001 | 0.535 | 0.090 | 0.177 | 0.111 | 0.005 | 0.001 |
| **Skip meals** | |  |  |  |  |  | 0.365 |  |  |  |  |  |  |
|  | No | 720 (76.2) | 175 (72) | 185 (74.6) | 169 (77.5) | 191 (80.9) |  |  |  |  |  |  |  |
|  | As before | 169 (17.9) | 50 (20.6) | 46 (18.5) | 37 (17) | 36 (15.3) |  |  |  |  |  |  |  |
|  | Yes | 56 (5.9) | 18 (7.4) | 17 (6.9) | 12 (5.5) | 9 (3.8) |  |  |  |  |  |  |  |
| **Number of meals done** | |  |  |  |  |  | 0.471 |  |  |  |  |  |  |
|  | 1 | 5 (0.5) | 0 (0) | 1 (0.4) | 2 (0.9) | 2 (0.8) |  |  |  |  |  |  |  |
|  | 2 | 25 (2.6) | 2 (0.8) | 7 (2.8) | 7 (3.2) | 9 (3.8) |  |  |  |  |  |  |  |
|  | 3 | 331 (35.0) | 85 (35) | 82 (33.1) | 81 (37.2) | 83 (35.2) |  |  |  |  |  |  |  |
|  | 4 | 367 (38.8) | 106 (43.6) | 98 (39.5) | 84 (38.5) | 79 (33.5) |  |  |  |  |  |  |  |
|  | 5 | 178 (18.8) | 39 (16) | 51 (20.6) | 37 (17) | 51 (21.6) |  |  |  |  |  |  |  |
|  | 6 | 39 (4.1) | 11 (4.5) | 9 (3.6) | 7 (3.2) | 12 (5.1) |  |  |  |  |  |  |  |
| **Quantity and quality of foods** | |  |  |  |  |  | 0.635 |  |  |  |  |  |  |
|  | Less and better | 141 (14.9) | 34 (29.8) | 42 (37.8) | 36 (39.1) | 29 (31.9) |  |  |  |  |  |  |  |
|  | More and better | 181 (19.2) | 43 (37.7) | 56 (50.5) | 36 (39.1) | 46 (50.5) |  |  |  |  |  |  |  |
|  | More and worse | 85 (9.0) | 31 (27.2) | 25 (22.5) | 17 (18.5) | 12 (13.2) |  |  |  |  |  |  |  |
|  | Less and worse | 19 (2.0) | 6 (5.3) | 6 (5.4) | 3 (3.3) | 4 (4.4) |  |  |  |  |  |  |  |
| **Fresh or frozen product** | |  |  |  |  |  | 0.198 |  |  |  |  |  |  |
|  | Fresh | 420 (44.4) | 94 (38.7) | 111 (44.8) | 97 (44.5) | 118 (50) |  |  |  |  |  |  |  |
|  | Frozen | 19 (2.0) | 5 (2.1) | 7 (2.8) | 2 (0.9) | 5 (2.1) |  |  |  |  |  |  |  |
|  | Both | 506 (53.5) | 144 (59.3) | 130 (52.4) | 119 (54.6) | 113 (47.9) |  |  |  |  |  |  |  |
| **Proximity product** | |  |  |  |  |  | 0.016 | 0.576 | 0.635 | 0.415 | 0.488 | 0.282 | 0.006 |
|  | No | 39 (4.1) | 8 (3.7) | 14 (6.1) | 8 (4) | 9 (4) | 0.585 |  |  |  |  |  |  |
|  | Sometimes | 235 (24.9) | 71 (33.2) | 65 (28.4) | 51 (25.5) | 48 (21.2) | 0.039 | 0.078 | 0.287 | 0.324 | 0.153 | 0.182 | 0.018 |
|  | Yes | 595 (63.0) | 135 (63.1) | 150 (65.5) | 141 (70.5) | 169 (74.8) | 0.039 | 0.333 | 0.403 | 0.342 | 0.242 | 0.096 | 0.030 |
| **Fast or convenience foods** | |  |  |  |  |  | 0.559 |  |  |  |  |  |  |
|  | No | 799 (84.6) | 202 (83.1) | 211 (85.1) | 182 (83.5) | 204 (86.4) |  |  |  |  |  |  |  |
|  | As before | 99 (10.5) | 24 (9.9) | 24 (9.7) | 26 (11.9) | 25 (10.6) |  |  |  |  |  |  |  |
|  | Yes | 47 (5.0) | 17 (7) | 13 (5.2) | 10 (4.6) | 7 (3) |  |  |  |  |  |  |  |
| **Time spent cooking** | |  |  |  |  |  | 0.006 | 0.597 | 0.329 | 0.353 | 0.738 | 0.006 | 0.054 |
|  | No | 123 (13.0) | 26 (10.7) | 25 (10.1) | 27 (12.4) | 45 (19.1) | 0.013 | 0.469 | 0.595 | 0.129 | 0.560 | 0.024 | 0.035 |
|  | As before | 183 (19.4) | 45 (18.5) | 38 (15.3) | 48 (22) | 52 (22) | 0.190 |  |  |  |  |  |  |
|  | Yes | 639 (67.6) | 172 (70.8) | 185 (74.6) | 143 (65.6) | 139 (58.9) | 0.002 | 0.198 | 0.085 | 0.234 | 0.255 | <0.001 | 0.020 |
| **Type of Cooking** | |  |  |  |  |  |  |  |  |  |  |  |  |
|  | Fried | 259 (27.4) | 67 (8.5) | 77 (9.8) | 63 (9.3) | 52 (7.7) | 0.150 | 0.540 | 0.570 | 0.258 | 0.416 | 0.092 | 0.335 |
|  | Microwave oven | 130 (13.8) | 51 (6.5) | 36 (4.6) | 23 (3.4) | 20 (3) | <0.001 | 0.112 | 0.236 | 0.276 | 0.010 | 0.100 | <0.001 |
|  | Griddle | 744 (78.7) | 201 (25.5) | 193 (24.5) | 175 (25.7) | 175 (25.8) | 0.128 |  |  |  |  |  |  |
|  | Boiled or steamed | 558 (59.0) | 148 (18.8) | 147 (18.7) | 139 (20.4) | 124 (18.3) | 0.090 |  |  |  |  |  |  |
|  | Papillote | 39 (4.1) | 3 (0.4) | 12 (1.5) | 15 (2.2) | 9 (1.3) | 0.021 | 0.087 | 0.406 | 0.285 | 0.012 | 0.372 | 0.232 |
|  | Roast or oven | 646 (68.4) | 182 (23.1) | 177 (22.5) | 144 (21.1) | 143 (21.1) | 0.005 | 0.218 | 0.337 | 0.250 | 0.093 | 0.039 | 0.006 |
|  | Casseroles or stews | 526 (55.7) | 130 (16.5) | 136 (17.3) | 114 (16.7) | 146 (21.6) | 0.157 |  |  |  |  |  |  |
|  | Others | 32 (3.4) | 7 (0.9) | 9 (1.1) | 8 (1.2) | 8 (1.2) | 0.962 |  |  |  |  |  |  |
| **Fried foods^1^** | |  |  |  |  |  | 0.415 |  |  |  |  |  |  |
|  | No | 708 (74.9) | 176 (72.4) | 178 (71.8) | 169 (77.5) | 185 (78.4) |  |  |  |  |  |  |  |
|  | As before | 142 (15.0) | 40 (16.5) | 38 (15.3) | 31 (14.2) | 33 (14) |  |  |  |  |  |  |  |
|  | Yes | 95 (10.1) | 27 (11.1) | 32 (12.9) | 18 (8.3) | 18 (7.6) |  |  |  |  |  |  |  |
| **Sociability**^2^ | |  |  |  |  |  | <0.001 | 0.008 | 0.255 | 0.005 | 0.009 | <0.001 | 0.433 |
|  | No | 77 (8.1) | 24 (10.5) | 25 (10.7) | 13 (6.3) | 15 (7.1) | <0.001 | 0.005 | 0.415 | 0.009 | <0.001 | 0.008 | 0.471 |
|  | As before | 327 (34.6) | 97 (42.5) | 64 (27.4) | 62 (30.2) | 104 (49.3) | 0.242 |  |  |  |  |  |  |
|  | Yes | 474 (50.2) | 107 (46.9) | 145 (62) | 130 (63.4) | 92 (43.6) | <0.001 | <0.001 | 0.287 | 0.008 | 0.015 | 0.005 | 0.177 |
| **Physical Activity** | |  |  |  |  |  | <0.001 | 0.144 | 0.715 | 0.651 | 0.005 | 0.476 | <0.001 |
|  | As before | 148 (15.7) | 42 (17.3) | 42 (16.9) | 29 (13.3) | 35 (14.8) | 0.608 |  |  |  |  |  |  |
|  | Higher | 218 (23.1) | 82 (33.7) | 56 (22.6) | 42 (19.3) | 38 (16.1) | <0.001 | 0.016 | 0.396 | 0.224 | <0.001 | 0.132 | 0.005 |
|  | Lower | 520 (55.0) | 107 (44) | 134 (54) | 130 (59.6) | 149 (63.1) | <0.001 | 0.066 | 0.245 | 0.252 | 0.005 | 0.076 | <0.001 |
|  | Sedentarism | 59 (6.2) | 12 (4.9) | 16 (6.5) | 17 (7.8) | 14 (5.9) | 0.645 |  |  |  |  |  |  |
| **Frequency of Physical Activity** | |  |  |  |  |  | 0.094 |  |  |  |  |  |  |
|  | **30 to 60 minutes** |  |  |  |  |  |  |  |  |  |  |  |  |
|  | Every day or almost | 289 (30.6) | 93 (38.3) | 66 (26.6) | 52 (23.9) | 78 (33.1) | 0.003 | 0.020 | 0.282 | 0.074 | 0.006 | 0.206 | 0.254 |
|  | 3-4 times a week | 209 (22.1) | 48 (19.8) | 58 (23.4) | 54 (24.8) | 49 (20.8) | 0.539 |  |  |  |  |  |  |
|  | Once to twice a week | 142 (15.0) | 38 (15.6) | 39 (15.7) | 34 (15.6) | 31 (13.1) | 0.830 |  |  |  |  |  |  |
|  | Once a week | 99 (10.5) | 25 (10.3) | 29 (11.7) | 25 (11.5) | 20 (8.5) | 0.651 |  |  |  |  |  |  |
|  | Sedentarism | 206 (21.8) | 39 (16) | 56 (22.6) | 53 (24.3) | 58 (24.6) | 0.083 |  |  |  |  |  |  |
|  | **> 60 minutes** |  |  |  |  |  | 0.203 |  |  |  |  |  |  |
|  | Every day or almost | 83 (8.8) | 27 (11.1) | 14 (5.6) | 17 (7.8) | 25 (10.6) | 0.116 |  |  |  |  |  |  |
|  | 3-4 times a week | 65 (6.9) | 23 (9.5) | 12 (4.8) | 15 (6.9) | 15 (6.4) | 0.236 |  |  |  |  |  |  |
|  | Once to twice a week | 100 (10.6) | 30 (12.3) | 28 (11.3) | 22 (10.1) | 20 (8.5) | 0.553 |  |  |  |  |  |  |
|  | Once a week | 118 (12.5) | 33 (13.6) | 31 (12.5) | 29 (13.3) | 25 (10.6) | 0.759 |  |  |  |  |  |  |
|  | Sedentarism | 579 (61.3) | 130 (53.5) | 163 (65.7) | 135 (61.9) | 151 (64) | 0.029 | 0.024 | 0.535 | 0.592 | 0.154 | 0.380 | 0.063 |
| **Sleep quality** | |  |  |  |  |  | 0.320 |  |  |  |  |  |  |
|  | Worse | 323 (34.2) | 81 (33.3) | 94 (37.9) | 65 (29.8) | 83 (35.2) |  |  |  |  |  |  |  |
|  | As before | 469 (49.6) | 119 (49) | 122 (49.2) | 109 (50) | 119 (50.4) |  |  |  |  |  |  |  |
|  | Better | 153 (16.2) | 43 (17.7) | 32 (12.9) | 44 (20.2) | 34 (14.4) |  |  |  |  |  |  |  |
| **Sleep hours** | |  |  |  |  |  | <0.001 | <0.001 | 0.440 | 0.551 | 0.005 | 0.012 | 0.008 |
|  | >8 hours | 104 (11.0) | 40 (16.5) | 33 (13.3) | 19 (8.7) | 12 (5.1) | <0.001 | 0.196 | 0.214 | 0.170 | 0.036 | 0.005 | <0.001 |
|  | 7-8 hours | 374 (39.6) | 120 (49.4) | 79 (31.9) | 78 (35.8) | 97 (41.1) | 0.001 | <0.001 | 0.213 | 0.266 | 0.010 | 0.085 | 0.121 |
|  | 6-7 hours | 292 (30.9) | 55 (22.6) | 90 (36.3) | 73 (33.5) | 74 (31.4) | 0.008 | 0.006 | 0.504 | 0.304 | 0.030 | 0.379 | 0.078 |
|  | 5-6 hours | 150 (15.9) | 22 (9.1) | 42 (16.9) | 43 (19.7) | 43 (18.2) | 0.007 | 0.028 | 0.587 | 0.623 | 0.006 | 0.400 | 0.010 |
|  | <5 hours | 25 (2.6) | 6 (2.5) | 4 (1.6) | 5 (2.3) | 10 (4.2) | 0.323 |  |  |  |  |  |  |
| **Smoking status** | |  |  |  |  |  | <0.001 | <0.001 | 0.234 | 0.090 | 0.005 | 0.363 | 0.008 |
|  | Current smoker | 180 (19.0) | 60 (24.7) | 40 (16.1) | 44 (20.2) | 36 (15.3) | 0.032 | 0.059 | 0.286 | 0.358 | 0.382 | 0.445 | 0.041 |
|  | Non-smoker | 483 (51.1) | 165 (67.9) | 126 (50.8) | 97 (44.5) | 95 (40.3) | <0.001 | 0.005 | 0.038 | 0.008 | 0.194 | 0.206 | <0.001 |
|  | Former smoker | 282 (29.8) | 18 (7.4) | 82 (33.1) | 77 (35.3) | 105 (44.5) | <0.001 | <0.001 | 0.339 | 0.057 | 0.005 | 0.018 | 0.008 |
| **Smoking pattern** | |  |  |  |  |  | <0.001 | <0.001 | 0.208 | 0.333 | 0.005 | 0.085 | 0.008 |
|  | As before | 87 (48.3) | 25 (41.7) | 18 (45) | 22 (50) | 22 (61.1) | 0.643 |  |  |  |  |  |  |
|  | More | 48 (26.7) | 12 (20) | 17 (42.5) | 11 (25) | 8 (22.2) | 0.387 |  |  |  |  |  |  |
|  | Less | 45 (25.0) | 23 (38.3) | 5 (12.5) | 11 (25) | 6 (16.7) | <0.001 | <0.001 | 0.140 | 0.174 | 0.005 | 0.015 | 0.008 |
| **Daily consumption of alcohol** | |  |  |  |  |  | <0.001 | <0.001 | 0.518 | 0.055 | 0.005 | 0.081 | 0.067 |
|  | No | 776 (77.9) | 212 (87.2) | 178 (71.8) | 157 (72) | 189 (80.1) |  |  |  |  |  |  |  |
|  | Yes | 209 (22.1) | 31 (12.8) | 70 (28.2) | 61 (28) | 47 (19.9) |  |  |  |  |  |  |  |
| **Daily consumption of sweetened beverages** | |  |  |  |  |  | 0.031 | 0.313 | 0.077 | 0.497 | 0.366 | 0.047 | 0.326 |
|  | No | 867 (91.7) | 221 (90.9) | 218 (87.9) | 205 (94) | 223 (94.5) |  |  |  |  |  |  |  |
|  | Yes | 78 (8.3) | 22 (9.1) | 30 (12.1) | 13 (6) | 13 (5.5) |  |  |  |  |  |  |  |

*Values are n (%). ¥ χ2-test for categorical variable. γ P-values were corrected for multiple comparisons following the Holm-Bonferroni’s method. ^1^ Fried food: Frying, the food is completely immersed in a deeper vessel of hot oil; ^2^ Eat together as a family.

**Supplementary** **Table 2.** **Changes in food intake and the Mediterranean diet pattern during the COVID-19 lockdown.**

| **Food Groups** |  |  | **< 33y. (Q1)** | **33-44y. (Q2)** | **44-53y. (Q3)** | **>53y. (Q4)** | **P-values were corrected for multiple comparisons γ** | | | | | | |
| --- | --- | --- | --- | --- | --- | --- | --- | --- | --- | --- | --- | --- | --- |
|  |  | **Spanish participants**  **N=945** | **N** | **N** | **N** | **N** | **P-value¥** | **Q1 vs Q2** | **Q2 vs Q3** | **Q3 vs Q4** | **Q1 vs Q3** | **Q2 vs Q4** | **Q1 vs Q4** |
| **Nuts** **(Servings/d)** | |  |  |  |  |  | 0.117 |  |  |  |  |  |  |
|  | ≥3 | 261 (27.6) * | 67 (27.6) | 74 (29.8) | 49 (22.5) | 71 (30.1) |  |  |  |  |  |  |  |
|  | 2-3 | 234 (24.8) | 62 (25.5) | 61 (24.6) | 57 (26.1) | 54 (22.9) |  |  |  |  |  |  |  |
|  | 1-2 | 338 (35.8) | 75 (30.9) | 84 (33.9) | 93 (42.7) | 86 (36.4) |  |  |  |  |  |  |  |
|  | 0 | 112 (11.9) | 39 (16) | 29 (11.7) | 19 (8.7) | 25 (10.6) |  |  |  |  |  |  |  |
| **Vegetables (Servings/d)** | |  |  |  |  |  | 0.001 | 0.317 | 0.241 | 0.293 | 0.077 | 0.208 | <0.001 |
|  | ≥2 | 302 (32.0) | 99 (40.7) | 83 (33.5) | 61 (28) | 59 (25) | 0.001 | 0.164 | 0.224 | 0.270 | 0.015 | 0.100 | <0.001 |
|  | 1-2 | 619 (65.5) | 136 (56) | 161 (64.9) | 148 (67.9) | 174 (73.7) | 0.001 | 0.076 | 0.282 | 0.195 | 0.030 | 0.089 | <0.001 |
|  | 0 | 24 (2.5) | 8 (3.3) | 4 (1.6) | 9 (4.1) | 3 (1.3) | 0.160 |  |  |  |  |  |  |
| **Fruits (Servings/d)** | |  |  |  |  |  | 0.005 | 0.114 | 0.454 | 0.365 | 0.118 | 0.164 | 0.070 |
|  | ≥3 | 70 (7.4) | 16 (6.6) | 17 (6.9) | 12 (5.5) | 25 (10.6) | 0.172 |  |  |  |  |  |  |
|  | 2-3 | 243 (25.7) | 71 (29.2) | 51 (20.6) | 56 (25.7) | 65 (27.5) | 0.144 |  |  |  |  |  |  |
|  | 1-2 | 565 (59.8) | 128 (52.7) | 162 (65.3) | 139 (63.8) | 136 (57.6) | 0.017 | 0.018 | 0.399 | 0.288 | 0.049 | 0.185 | 0.159 |
|  | 0 | 67 (7.1) | 28 (11.5) | 18 (7.3) | 11 (5) | 10 (4.2) | 0.009 | 0.255 | 0.382 | 0.425 | 0.044 | 0.293 | 0.012 |
| ***Sofrito* (Servings/d)** | |  |  |  |  |  | 0.363 |  |  |  |  |  |  |
|  | ≥ 2 | 44 (4.7) | 16 (6.6) | 10 (4) | 10 (4.6) | 8 (3.4) |  |  |  |  |  |  |  |
|  | 1-2 | 147 (15.6) | 38 (15.6) | 41 (16.5) | 34 (15.6) | 34 (14.4) |  |  |  |  |  |  |  |
|  | ≤ 1 | 650 (68.8) | 162 (66.7) | 161 (64.9) | 152 (69.7) | 175 (74.2) |  |  |  |  |  |  |  |
|  | Never | 104 (11.0) | 27 (11.1) | 36 (14.5) | 22 (10.1) | 19 (8.1) |  |  |  |  |  |  |  |
| **Legumes (Servings/w)** | |  |  |  |  |  | <0.001 | 0.039 | 0.281 | 0.105 | 0.006 | 0.096 | 0.079 |
|  | ≥3 | 47 (5.0) | 24 (9.9) | 11 (4.4) | 4 (1.8) | 8 (3.4) | <0.001 | 0.059 | 0.249 | 0.409 | <0.001 | 0.361 | 0.015 |
|  | 2-3 | 337 (35.7) | 89 (36.6) | 86 (34.7) | 72 (33) | 90 (38.1) | 0.684 |  |  |  |  |  |  |
|  | 1-2 | 546 (57.8) | 129 (53.1) | 142 (57.3) | 137 (62.8) | 138 (58.5) | 0.207 |  |  |  |  |  |  |
|  | 0 | 15 (1.6) | 1 (0.4) | 9 (3.6) | 5 (2.3) | 0 (0) | 0.004 | 0.054 | 0.490 | 0.096 | 0.234 | 0.012 | 0.507 |
| **Grains (Servings/d)** | |  |  |  |  |  | 0.606 |  |  |  |  |  |  |
|  | ≥3 | 25 (2.6) | 7 (2.9) | 5 (2) | 7 (3.2) | 6 (2.5) |  |  |  |  |  |  |  |
|  | 2-3 | 291 (30.8) | 76 (31.3) | 70 (28.2) | 60 (27.5) | 85 (36) |  |  |  |  |  |  |  |
|  | 1-2 | 595 (63.0) | 150 (61.7) | 166 (66.9) | 144 (66.1) | 135 (57.2) |  |  |  |  |  |  |  |
|  | 0 | 34 (3.6) | 10 (4.1) | 7 (2.8) | 7 (3.2) | 10 (4.2) |  |  |  |  |  |  |  |
| **Potatoes (Servings/w)** | |  |  |  |  |  | 0.464 |  |  |  |  |  |  |
|  | ≥3 | 83 (8.8) | 27 (11.1) | 22 (8.9) | 19 (8.7) | 15 (6.4) |  |  |  |  |  |  |  |
|  | 2-3 | 389 (41.2) | 101 (41.6) | 91 (36.7) | 98 (45) | 99 (41.9) |  |  |  |  |  |  |  |
|  | 1 | 415 (43.9) | 97 (39.9) | 120 (48.4) | 90 (41.3) | 108 (45.8) |  |  |  |  |  |  |  |
|  | 0 | 58 (6.1) | 18 (7.4) | 15 (6) | 11 (5) | 14 (5.9) |  |  |  |  |  |  |  |
| **Dairy products (Servings/d)** | |  |  |  |  |  | 0.011 | 0.229 | 0.641 | 0.885 | 0.018 | 0.712 | 0.035 |
|  | ≥4 | 21 (2.2) | 4 (1.6) | 7 (2.8) | 5 (2.3) | 5 (2.1) | 0.849 |  |  |  |  |  |  |
|  | 3-4 | 153 (16.2) | 59 (24.3) | 37 (14.9) | 28 (12.8) | 29 (12.3) | 0.001 | 0.024 | 0.527 | 0.477 | 0.005 | 0.559 | <0.001 |
|  | 1-2 | 629 (66.6) | 144 (59.3) | 159 (64.1) | 156 (71.6) | 170 (72) | 0.006 | 0.286 | 0.129 | 0.528 | 0.015 | 0.144 | 0.012 |
|  | ≤1 | 103 (10.9) | 22 (9.1) | 33 (13.3) | 24 (11) | 24 (10.2) | 0.482 |  |  |  |  |  |  |
|  | 0 | 38 (4.0) | 14 (5.8) | 12 (4.8) | 4 (1.8) | 8 (3.4) | 0.155 |  |  |  |  |  |  |
| **Type of dairy products** | |  |  |  |  |  |  |  |  |  |  |  |  |
|  | Fresh cheese | 169 (49.9) | 126 (18.9) | 117 (20.1) | 110 (21.9) | 114 (22.2) | 0.737 |  |  |  |  |  |  |
|  | Cured cheese | 550 (58.8) | 135 (20.2) | 148 (25.5) | 132 (26.2) | 135 (26.3) | 0.767 |  |  |  |  |  |  |
|  | Philadelphia cheese | 162 (17.3) | 162 (24.3) | 41 (7.1) | 41 (8.2) | 36 (7) | 0.892 |  |  |  |  |  |  |
|  | Natural yogurts^1^ | 614 (65.6) | 141 (21.1) | 172 (29.6) | 143 (28.4) | 158 (30.7) | 0.053 |  |  |  |  |  |  |
|  | Sugary yogurt^2^ | 102 (10.9) | 26 (3.9) | 28 (4.8) | 27 (5.4) | 21 (4.1) | 0.772 |  |  |  |  |  |  |
|  | Fruit yogurts | 164 (17.5) | 51 (7.6) | 45 (7.7) | 34 (6.8) | 34 (6.6) | 0.232 |  |  |  |  |  |  |
|  | Curd | 75 (8.0) | 21 (3.1) | 28 (4.8) | 13 (2.6) | 13 (2.5) | 0.071 |  |  |  |  |  |  |
|  | Petit Suisse | 13 (1.4) | 5 (0.7) | 2 (0.3) | 3 (0.6) | 3 (0.6) | 0.692 |  |  |  |  |  |  |
| **Eggs (Servings/w)** | |  |  |  |  |  | 0.106 |  |  |  |  |  |  |
|  | ≥3 | 144 (15.2) | 47 (19.3) | 45 (18.1) | 26 (11.9) | 26 (11) |  |  |  |  |  |  |  |
|  | 2-3 | 497 (52.6) | 119 (49) | 123 (49.6) | 119 (54.6) | 136 (57.6) |  |  |  |  |  |  |  |
|  | 1-2 | 288 (30.5) | 71 (29.2) | 76 (30.6) | 68 (31.2) | 73 (30.9) |  |  |  |  |  |  |  |
|  | 0 | 16 (1.7) | 6 (2.5) | 4 (1.6) | 5 (2.3) | 1 (0.4) |  |  |  |  |  |  |  |
| **Red Meat (Servings/w)** | |  |  |  |  |  | <0.001 | 0.005 | 0.799 | 0.069 | 0.008 | 0.056 | <0.001 |
|  | All days | 24 (2.5) | 4 (1.6) | 11 (4.4) | 7 (3.2) | 2 (0.8) | 0.057 |  |  |  |  |  |  |
|  | >3 | 107 (11.3) | 30 (12.3) | 28 (11.3) | 31 (14.2) | 18 (7.6) | 0.152 |  |  |  |  |  |  |
|  | 2-3 | 325 (34.4) | 61 (25.1) | 83 (33.5) | 77 (35.3) | 104 (44.1) | <0.001 | 0.076 | 0.373 | 0.069 | 0.054 | 0.047 | <0.001 |
|  | ≤1 | 386 (40.8) | 98 (40.3) | 105 (42.3) | 86 (39.4) | 97 (41.1) | 0.932 |  |  |  |  |  |  |
|  | 0 | 103 (10.9) | 50 (20.6) | 21 (8.5) | 17 (7.8) | 15 (6.4) | <0.001 | <0.001 | 0.464 | 0.562 | 0.005 | 0.559 | 0.008 |
| **Lean meat (Servings/w)** | |  |  |  |  |  | <0.001 | 0.051 | 0.134 | 0.049 | 0.056 | 0.179 | 0.006 |
|  | All days | 15 (1.6) | 4 (1.6) | 6 (2.4) | 0 (0) | 5 (2.1) | 0.168 |  |  |  |  |  |  |
|  | >3 | 139 (14.7) | 37 (15.2) | 44 (17.7) | 36 (16.5) | 22 (9.3) | 0.048 | 0.603 | 0.411 | 0.077 | 0.641 | 0.030 | 0.126 |
|  | 2-3 | 448 (47.4) | 111 (45.7) | 112 (45.2) | 112 (51.4) | 113 (47.9) | 0.534 |  |  |  |  |  |  |
|  | ≤1 | 292 (30.9) | 64 (26.3) | 78 (31.5) | 61 (28) | 89 (37.7) | 0.038 | 0.330 | 0.418 | 0.087 | 0.385 | 0.308 | 0.030 |
|  | 0 | 51 (5.4) | 27 (11.1) | 8 (3.2) | 9 (4.1) | 7 (3) | <0.001 | 0.005 | 0.630 | 0.710 | 0.016 | 0.539 | <0.001 |
| **Fish & seafood (Servings/w)** | | |  |  |  |  | <0.001 | 0.231 | 0.539 | 0.185 | 0.020 | 0.005 | <0.001 |
|  | All days | 6 (0.6) | 3 (1.2) | 2 (0.8) | 1 (0.5) | 0 (0) | 0.374 |  |  |  |  |  |  |
|  | >3 | 99 (10.5) | 20 (8.2) | 18 (7.3) | 21 (9.6) | 40 (16.9) | 0.002 | 0.407 | 0.535 | 0.059 | 0.587 | 0.006 | 0.015 |
|  | 2-3 | 423 (44.8) | 90 (37) | 109 (44) | 107 (49.1) | 117 (49.6) | 0.020 | 0.255 | 0.286 | 0.496 | 0.030 | 0.330 | 0.024 |
|  | ≤1 | 364 (38.5) | 104 (42.8) | 108 (43.5) | 82 (37.6) | 70 (29.7) | 0.006 | 0.469 | 0.304 | 0.168 | 0.278 | 0.006 | 0.010 |
|  | 0 | 53 (5.6) | 26 (10.7) | 11 (4.4) | 7 (3.2) | 9 (3.8) | 0.001 | 0.028 | 0.701 | 0.464 | 0.006 | 0.703 | 0.015 |
| **Water (Glasses/d)** | |  |  |  |  |  | <0.001 | 0.076 | 0.190 | 0.241 | 0.028 | 0.005 | <0.001 |
|  | ≥ 8 | 161 (17.0) | 54 (22.2) | 54 (21.8) | 31 (14.2) | 22 (9.3) | <0.001 | 0.496 | 0.067 | 0.135 | 0.070 | 0.005 | <0.001 |
|  | 6-8 | 357 (37.8) | 105 (43.2) | 81 (32.7) | 82 (37.6) | 89 (37.7) | 0.121 |  |  |  |  |  |  |
|  | ≤ 5 | 427 (45.2) | 84 (34.6) | 113 (45.6) | 105 (48.2) | 125 (53) | 0.001 | 0.032 | 0.320 | 0.323 | 0.010 | 0.175 | <0.001 |
| **Type of alcohol intake** | |  |  |  |  |  |  |  |  |  |  |  |  |
|  | Wine^3^ | 413 (43.7) | 71 (39.9) | 98 (43) | 105 (50.5) | 139 (5.7) | <0.001 | 0.030 | 0.037 | 0.028 | <0.001 | 0.008 | 0.005 |
|  | Beer | 384 (40.6) | 91 (51.1) | 116 (50.9) | 91 (43.8) | 86 (3.5) | 0.081 |  |  |  |  |  |  |
|  | Spirits | 33 (3.5) | 9 (5.1) | 4 (1.8) | 9 (4.3) | 11 (0.5) | 0.278 |  |  |  |  |  |  |
|  | Liquor | 47 (5.0) | 7 (3.9) | 10 (4.4) | 3 (1.4) | 7 (0.3) | 0.397 |  |  |  |  |  |  |
| **Sweetened Beverages (Servings/d)** | |  |  |  |  |  | 0.052 |  |  |  |  |  |  |
|  | ≥ 2 | 32 (3.4) | 4 (1.6) | 17 (6.9) | 4 (1.8) | 7 (3) | 0.004 | 0.018 | 0.035 | 0.536 | 0.500 | 0.140 | 0.588 |
|  | 1 | 49 (5.2) | 10 (4.1) | 12 (4.8) | 14 (6.4) | 13 (5.5) | 0.718 |  |  |  |  |  |  |
|  | < 1 | 125 (13.2) | 27 (11.1) | 33 (13.3) | 37 (17) | 28 (11.9) | 0.262 |  |  |  |  |  |  |
|  | Sporadically | 242 (25.6) | 59 (24.3) | 64 (25.8) | 58 (26.6) | 61 (25.8) | 0.949 |  |  |  |  |  |  |
|  | Never | 497 (52.6) | 143 (58.8) | 122 (49.2) | 105 (48.2) | 127 (53.8) | 0.073 |  |  |  |  |  |  |
| **Juices (≥1 / d)** | |  |  |  |  |  | 0.451 |  |  |  |  |  |  |
|  | No | 676 (71.5) | 178 (73.3) | 175 (70.6) | 157 (72) | 166 (70.3) |  |  |  |  |  |  |  |
|  | Both | 27 (2.9) | 7 (2.9) | 7 (2.8) | 9 (4.1) | 4 (1.7) |  |  |  |  |  |  |  |
|  | Natural | 191 (20.2) | 40 (16.5) | 52 (21) | 43 (19.7) | 56 (23.7) |  |  |  |  |  |  |  |
|  | Commercial | 51 (5.4) | 18 (7.4) | 14 (5.6) | 9 (4.1) | 10 (4.2) |  |  |  |  |  |  |  |

*Values are n (%). ¥ χ2-test for categorical variable. γ P-values were corrected for multiple comparisons following the Holm-Bonferroni’s method.  ^1^Whole or skim plain yogurts; ^2^Sweetened, flavoured and chocolate yogurts; and ^3^White, red, rosé or sparkling wine. D = day; W = week.

**Supplementary Table 3.** **Associations between MeDiet pattern and weight gain in regarding to the COVID-19 confinement**

|  | **Crude Model^1^** | **Adjusted Model^2^** |
| --- | --- | --- |
|  | **OR (95% CI)** | **OR (95% CI)** |
| **Olive oil (sv/d)** | N= 312 (33%) | |
| Unknown | **Ref.** | **Ref.** |
| No | **0.53 (0.33 to 0.88)** | **0.54 (0.32 to 0.91)** |
| Yes | 0.81 (0.48 to 1.35) | 0.89 (0.51 to 1.53) |
| **Other oils (sv/d)** | N= 312 (33%) | |
| Unknown | **Ref.** | **Ref.** |
| No | 0.6 (0.32 to 1.12) | 0.57 (0.29 to 1.11) |
| Yes | 1.97 (0.92 to 4.22) | 2.09 (0.92 to 4.74) |
| **Nuts (sv/d)** | N= 312 (33%) | |
| No | **Ref.** | **Ref.** |
| Yes | 1.33 (1 to 1.78) | 1.33 (0.98 to 1.81) |
| **Nuts (sv/d)** | N= 312 (33%) | |
| 0 | **Ref.** | **Ref.** |
| 1-2 | 1.22 (0.77 to 1.93) | 1.07 (0.66 to 1.74) |
| 2-3 | 1.18 (0.72 to 1.91) | 1.12 (0.67 to 1.88) |
| >3 | 1.04 (0.64 to 1.68) | 1.03 (0.62 to 1.72) |
| **Vegetables (sv/d)** | N= 312 (33%) | |
| No | **Ref.** | **Ref.** |
| Yes | 0.77 (0.57 to 1.04) | 0.81 (0.59 to 1.91) |
| **Vegetables (sv/d)** | N= 312 (33%) | |
| 0 | **Ref.** | **Ref.** |
| 1-2 | 0.63 (0.28 to 1.44) | 0.61 (0.25 to 1.46) |
| ≥2 | 0.47 (0.2 to 1.08) | 0.47 (0.19 to 1.16) |
| ***Sofrito* (sv/d)** | N= 312 (33%) | |
| No | **Ref.** | **Ref.** |
| Yes | 1.17 (0.87 to 1.57) | 1.32 (0.96 to 1.8) |
| **Fresh Fruit (sv/d)** | N= 312 (33%) | |
| No | **Ref.** | **Ref.** |
| Yes | 1.14 (0.85 to 1.54) | 1.17 (0.86 to 1.6) |
| **Fresh Fruit (sv/d)** | N= 312 (33%) | |
| 0 | **Ref.** | **Ref.** |
| 1-2 | 0.64 (0.38 to 1.08) | 0.58 (0.33 to 1.01) |
| 2-3 | **0.56 (0.32 to 0.98)** | 0.58 (0.32 to 1.05) |
| ≥3 | **0.48 (0.23 to 0.97)** | **0.44 (0.21 to 0.95)** |
| **Legumes (sv/wk)** | N= 312 (33%) | |
| No | **Ref.** | **Ref.** |
| Yes | 1.09 (0.8 to 1.5) | 1.15 (0.82 to 1.6) |
| **Grains (sv/d)** | N= 312 (33%) | |
| No | **Ref.** | **Ref.** |
| Yes | **1.59 (1.17 to 2.17)** | **1.65 (1.19 to 2.29)** |
| **Whole grains (sv/d)** | N= 312 (33%) | |
| No | **Ref.** | **Ref.** |
| Yes | 0.98 (0.66 to 1.45) | 1.04 (0.68 to 1.58) |
| **Potatoes (sv/wk)** | N= 312 (33%) | |
| No | **Ref.** | **Ref.** |
| Yes | **1.51 (1.13 to 2.03)** | **1.6 (1.17 to 2.19)** |
| **Dairy products (sv/d)** | N= 312 (33%) | |
| No | **Ref.** | **Ref.** |
| Yes | **1.34 (0.98 to 1.84)** | **1.45 (1.04 to 2.02)** |
| **Eggs (sv./wk)** | N= 312 (33%) | |
| No | **Ref.** | **Ref.** |
| Yes | **1.47 (1.09 to 1.98)** | **1.47 (1.07 to 2.02)** |
| **Red meat (sv/wk)** | N= 312 (33%) | |
| No | **Ref.** | **Ref.** |
| Yes | **2.41 (1.67 to 3.47)** | **2.52 (1.7 to 3.73)** |
| **Lean meat (sv/wk)** | N= 312 (33%) | |
| No | **Ref.** | **Ref.** |
| Yes | 1.30 (0.87 to 1.95) | 1.32 (0.86 to 2.02) |
| **Fish and seafood (sv/w) (srv/wk)** | N= 62 (19.9%) | |
| No | **Ref.** | **Ref.** |
| Yes | 1.12 (0.79 to 1.59) | 1.30 (0.89 to 1.88) |
| **Alcohol (d)** | N= 312 (33%) | |
| No | **Ref.** | **Ref.** |
| Yes | **1.54 (1.12 to 2.11)** | **1.53 (1.09 to 2.15)** |
| **Sweetened beverages (d)** | N= 312 (33%) | |
| No | **Ref.** | **Ref.** |
| Yes | 1.55 (0.96 to 2.48) | 1.55 (0.96 to 2.48) |
| **Juices (≥1 /d)** | N= 312 (33%) | |
| No | **Ref.** | **Ref.** |
| Yes | 1.05 (0.78 to 1.42) | 1.09 (0.79 to 1.5) |
| **Homemade pastries** | N= 312 (33%) | |
| As before | **Ref.** | **Ref.** |
| No | 0.98 (0.6 to 1.61) | 0.98 (0.59 to 1.66) |
| Yes | **3.66 (1.97 to 6.81)** | **3.77 (1.95 to 7.27)** |
| **Non-homemade pastries** | N= 312 (33%) | |
| As before | **Ref.** | **Ref.** |
| No | 0.92 (0.58 to 1.44) | 0.94 (0.58 to 1.51) |
| Yes | 1.48 (0.97 to 2.25) | **1.59 (1.02 to 2.48)** |

^1^ Univariate regression model (Model 1); ^2^ Multivariate-adjusted model (Model 2). Model 2 adjusted by: sex (men and women), educational level (Postgraduate; University; High school; Primary and other studies), housing during confinement (with or without children; with elderly; and children and elderly; residence or shared flat) and type of AP (high, medium, low). ORs of the association between each studied variable related to eating and lifestyle habits and weight gain change were mutually adjusted by each other. Statistically significant ORs are highlighted in bold. d: day; sv: serving; wk: week.
