# Supplementary material for: Changes in Spanish lifestyle and dietary habits during the COVID-19 lockdown
Source: Eur J Nutr. 2022 Feb 7;61(5):2417–34. doi: 10.1007/s00394-022-02814-1 (PMC9279186; doi:10.1007/s00394-022-02814-1)
Supplement: Supplementary file 2 — Supplementary file2 (PDF 92 KB) [file 394_2022_2814_MOESM2_ESM.pdf]

Dietary habits and lifestyle during the confinement because the pandemic COVID-19

Fields marked with * are mandatory.

1 Information

Due to the situation produced by COVID-19, the Mediterranean Diet Foundation (https://dietamediterranea.com/), a partner of the Interreg-MD.net project (https://mdnet.interreg-med.eu/), proposes that a study be carried out in accordance with Regulation (EU) 2016/679 of the European Parliament and of the Council of April 27 2016 on the protection of individuals regarding the processing of personal data and on the free movement of data, Organic Law 3/2018 of December 5th on the Protection of Personal Data and the Ensuring of Digital Rights, on the eating habits and lifestyle of the Spanish population in the face of this state of emergency.

Your full voluntary participation in the study can help you to better understand your current eating habits and lifestyle. As you are well aware, the Mediterranean diet helps prevent diseases such as stroke or other cardiovascular diseases, as well as other chronic conditions. The information obtained from this study is expected to contribute to a better understanding of the effect of the Mediterranean Diet on cardiovascular disease, long-term weight loss and other chronic diseases.

This questionnaire targets the entire population, of the age group, who voluntarily and anonymously wants to participate. Therefore, we would **be very grateful for your support** of our research. We would also like to inform you that this data, as part of a database at European level, where other research groups are also doing similar surveys to their fellow citizens.

If you have any questions about the survey or about this research project, please do not hesitate to contact the project researchers at any time: Rosa Casas (rcasas1@clinic.cat) and Blanca Raidó (braido@fdmed.org). This project is led by Dr Ramon Estruch (restruch@clinic.cat), Senior Consultant in the Internal Medicine Department at the Hospital Clinic of Barcelona and the Scientific Chairman of the Mediterranean Diet Foundation.

If you are replying to this questionnaire, it is tacitly understood that you have understood the purpose of this study and therefore accept to participate.

Please answer all questions. It will take you no more than 12 minutes.

Thank you for your time!

2 Basic data

* 2.1 Sex

- Female
- Male
- Other
- Prefer not to say

* 2.2 Age

Only values of at least 18 are allowed

[ ] years

* 2.3 Country of origin

- Afghanistan
- Albania
- Algeria
- Andorra
- Angola
- Antigua and Barbuda
- Argentina
- Armenia
- Australia
- Austria
- Azerbaijan
- Bahamas
- Bahrain
- Bangladesh
- Barbados
- Belarus
- Belgium
- Belize
- Benin
- Bhutan
- Bolivia
- Bosnia and Herzegovina
- Botswana
- Brazil
- Brunei Darussalam
- Bulgaria
- Burkina Faso
- Burundi
- Cambodia
- Cameroon
- Canada

- Cape Verde
- Central African Republic
- Chad
- Chile
- China
- Colombia
- Comoros
- Congo
- Costa Rica
- Côte d’Ivoire
- Croatia
- Cuba
- Cyprus
- Czechia
- Democratic Republic of the Congo
- Denmark
- Djibouti
- Dominica
- Dominican Republic
- Ecuador
- Egypt
- Emirates
- Equatorial Guinea
- Eritrea
- Estonia
- Eswatini
- Ethiopia
- Fiji
- Finland
- France
- Gabon
- Gambia
- Georgia
- Germany
- Ghana
- Great Britain
- Greece
- Grenada
- Guatemala
- Guinea
- Guinea-Bissau
- Guyana
- Haiti
- Honduras
- Hungary
- Iceland
- India

- Indonesia
- Iran
- Iraq
- Ireland
- Israel
- Italy
- Jamaica
- Japan
- Jordan
- Kazakhstan
- Kenya
- Kiribati
- Korea, Republic of
- Kuwait
- Kyrgyzstan
- Laos
- Latvia
- Lebanon
- Lesotho
- Liberia
- Libya
- Liechtenstein
- Lithuania
- Luxembourg
- Madagascar
- Malawi
- Malaysia
- Maldives
- Mali
- Malta
- Marshall Islands
- Mauritania
- Mauritius
- Mexico
- Micronesia
- Monaco
- Mongolia
- Montenegro
- Morocco
- Mozambique
- Myanmar/Burma
- Namibia
- Nauru
- Nepal
- Netherlands
- New Zealand
- Nicaragua

- Niger
- Nigeria
- North Korea (Democratic Republic of North Korea)
- North Macedonia
- Norway
- Oman
- Pakistan
- Palau
- Panama
- Papua New Guinea
- Paraguay
- Peru
- Philippines
- Poland
- Portugal
- Qatar
- Republic of Moldova
- Romania
- Russia (Russian Federation)
- Rwanda
- Saint Kitts and Nevis
- Saint Vincent and Grenadines
- Salvador
- Samoa
- San Marino
- Santa Lucia
- São Tomé and Principe
- Saudi Arabia
- Senegal
- Serbia.
- Seychelles
- Sierra Leone
- Singapore
- Slovakia
- Slovenia
- Solomon Islands
- Somalia
- South Africa
- South Sudan
- Spain
- Sri Lanka
- Sudan
- Surinam
- Sweden
- Switzerland
- Syrian Arab Republic
- Tajikistan

- Tanzania
- Thailand
- The United States of America
- Timor Leste
- Togo
- Tonga
- Trinidad and Tobago
- Tunisia
- Turkey
- Turkmenistan
- Tuvalu
- Uganda
- Ukraine
- Uruguay
- Uzbekistan
- Vanuatu
- Venezuela
- Vietnam
- Yemen
- Zambia
- Zimbabwe

* 2.4 My regular residence is located in:

- A rural area
- An urban area

* 2.5 Marital status

- Single
- Married
- Common law
- Widowed
- Divorced / Separated
- Prefer not to say

* 2.6 Do you have any children?

- Yes
- No

* 2.7 What is your highest level of education achieved?

- No educational level
- Primary
- Secondary
- College or Trade School
- Degree/Graduated
- Doctorate, Master’s degree, post graduate
- Other training studies

* 2.8 What is your employment status?

- ○ Student
- ○ Employed or dependent public or private sector
- ○ Autonomous or independent
- ○ Recipient of undeclared income
- ○ Benefit
- ○ Temporary Employment Regulation
- ○ Retired
- ○ Prefer not to say

* 2.9 How do you continue to do your job?

between 1 and 9 choices

- ☐ Virtually
- ☐ I (almost) all work in person
- ☐ Half of the work in person and the other half virtually
- ☐ I (almost) all work virtually
- ☐ Still employed, but I am at home (almost) without working virtually
- ☐ Receiving a subsidy
- ☐ Not working
- ☐ Not working or studying
- ☐ Prefer not to say

* 2.10 On the basis of your household economy, what option represents you most?

- ○ Low income
- ○ Average Income
- ○ High income

3 Actual situation

* 3.1 How many days have you been in confinement?

[ ] days

* 3.2 I am confined in:

- ○ Afghanistan
- ○ Albania
- ○ Algeria
- ○ Andorra
- ○ Angola
- ○ Antigua and Barbuda
- ○ Argentina
- ○ Armenia
- ○ Australia
- ○ Austria
- ○ Azerbaijan

- Bahamas
- Bahrain
- Bangladesh
- Barbados
- Belarus
- Belgium
- Belize
- Benin
- Bhutan
- Bolivia
- Bosnia and Herzegovina
- Botswana
- Brazil
- Brunei Darussalam
- Bulgaria
- Burkina Faso
- Burundi
- Cambodia
- Cameroon
- Canada
- Cape Verde
- Central African Republic
- Chad
- Chile
- China
- Colombia
- Comoros
- Congo
- Costa Rica
- Côte d'Ivoire
- Croatia
- Cuba
- Cyprus
- Czechia
- Democratic Republic of the Congo
- Denmark
- Djibouti
- Dominica
- Dominican Republic
- Ecuador
- Egypt
- Emirates
- Equatorial Guinea
- Eritrea
- Estonia
- Eswatini
- Ethiopia

- Fiji
- Finland
- France
- Gabon
- Gambia
- Georgia
- Germany
- Ghana
- Great Britain
- Greece
- Grenada
- Guatemala
- Guinea
- Guinea-Bissau
- Guyana
- Haiti
- Honduras
- Hungary
- Iceland
- India
- Indonesia
- Iran
- Iraq
- Ireland
- Israel
- Italy
- Jamaica
- Japan
- Jordan
- Kazakhstan
- Kenya
- Kiribati
- Korea, Republic of
- Kuwait
- Kyrgyzstan
- Laos
- Latvia
- Lebanon
- Lesotho
- Liberia
- Libya
- Liechtenstein
- Lithuania
- Luxembourg
- Madagascar
- Malawi
- Malaysia

- Maldives
- Mali
- Malta
- Marshall Islands
- Mauritania
- Mauritius
- Mexico
- Micronesia
- Monaco
- Mongolia
- Montenegro
- Morocco
- Mozambique
- Myanmar/Burma
- Namibia
- Nauru
- Nepal
- Netherlands
- New Zealand
- Nicaragua
- Niger
- Nigeria
- North Korea (Democratic Republic of North Korea)
- North Macedonia
- Norway
- Oman
- Pakistan
- Palau
- Panama
- Papua New Guinea
- Paraguay
- Peru
- Philippines
- Poland
- Portugal
- Qatar
- Republic of Moldova
- Romania
- Russia (Russian Federation)
- Rwanda
- Saint Kitts and Nevis
- Saint Vincent and Grenadines
- Salvador
- Samoa
- San Marino
- Santa Lucia
- São Tomé and Principe

- Saudi Arabia
- Senegal
- Serbia.
- Seychelles
- Sierra Leone
- Singapore
- Slovakia
- Slovenia
- Solomon Islands
- Somalia
- South Africa
- South Sudan
- Spain
- Sri Lanka
- Sudan
- Surinam
- Sweden
- Switzerland
- Syrian Arab Republic
- Tajikistan
- Tanzania
- Thailand
- The United States of America
- Timor Leste
- Togo
- Tonga
- Trinidad and Tobago
- Tunisia
- Turkey
- Turkmenistan
- Tuvalu
- Uganda
- Ukraine
- Uruguay
- Uzbekistan
- Vanuatu
- Venezuela
- Vietnam
- Yemen
- Zambia
- Zimbabwe

* 3.3 I am confined:

between 1 and 8 choices

- [ ] Alone
- [ ] Family home with children
- [ ] Family home without children

- [ ] Family home with elderly
- [ ] Shared apartment
- [ ] Hospital
- [ ] Assisted living
- [ ] Prefer not to say

* 3.4 Do you think you have increased in weight since? (compared to before the confinement)

- ○ Increase
- ○ Decreased
- ○ No change
- ○ Do not know

4 Sleeping and sleeping sleep

* 4.1 How would you define your current quality of sleep? (compared to before the confinement)

- ○ Much worse
- ○ Same
- ○ Much better

* 4.2 How long do you sleep most of the nights during the confinement?

- ○ Less than 5 hours
- ○ Between 5 and 6 hours
- ○ Between 6 and 7 hours
- ○ Between 7 and 8 hours
- ○ Over 8 hours

5 Tobacco

* 5.1 How would you define your relationship with tobacco now? (comparing it to before the confinement)

- ○ I smoke much more.
- ○ Same as before
- ○ I smoke much less
- ○ I'm an ex-smoker
- ○ Never smoked

6 Physical activity and sport

* 6.1 How would you define your physical activity now? (comparing it to before confinement)

- ○ It increased
- ○ It decreased
- ○ No change.
- ○ I am not physically active, now or before

* 6.2 During the confinement, I am physically active during 30-60 minutes per day:

- Never
- Once a week
- One to twice a week
- Between 3 and 4 times a week
- Every day or almost every day of the week

* 6.3 During confinement, I am physically active fo more than 60 minutes per day:

- Never
- Once a week
- One to twice a week
- Between 3 and 4 times a week
- Every day or almost every day of the week

7 Dietary habits

* 7.1 How would you define your relationship with food? (comparing it to before confinement)

- Has changed
- Not changed
- Do not know

7.2 If you replied that it has changed, in what way?

- I eat more and worse quality
- I eat more and better quality
- I eat less and worse quality
- I eat less and worse quality
- Do not know

* 7.3 During confinement, how many meals do you usually have? (select all meals you have)

between 1 and 6 choices

- Breakfast
- Before breakfast snack
- Lunch
- Afternoon snack
- Dinner
- After dinner snack

* 7.4 In your opinion, do you snack more than before confinement?

- Yes
- No
- Do not know

* 7.5 Describe your snacks:

between 1 and 10 choices

- Nuts, roasted or salted
- Natural nuts

- ☐ Fried nuts
- ☐ Fried snacks
- ☐ Popcorn
- ☐ Potato chips/crisps
- ☐ Chocolate
- ☐ Cereal cakes
- ☐ Industrial baked goods (muffins, biscuits, cookies, croissants, donuts, etc.)
- ☐ Savoury snacks
- ☐ Fruit or vegetables
- ☐ Dairy
- ☐ I don’t snack

* 7.6 Have you skipped any main meals during confinement?

- ◯ Yes, but I never did before confinement
- ◯ Yes, but I did before confinement
- ◯ No

* 7.7 Do you think you are eating more than usual during this confinement?

- ◯ Yes
- ◯ No
- ◯ Same quantities as always

7.8 If you live with someone, have your habits of eating together changed during this period?

- ◯ Yes, now we eat more together
- ◯ No, we already ate most meals together
- ◯ No, we do not eat most meals together

* 7.9 We want to assess how much your eating habits are close to the Mediterranean diet. On a scale from 0 to 10, how would you rate your diet, being 0 very bad and 10 excellent?

Only values between 0 and 10 are allowed

8 Grocery shopping

* 8.1 During confinement, do you buy fresh or frozen products?

- ◯ Fresh
- ◯ Frozen
- ◯ Fresh and frozen without distinction

* 8.2 During confinement, has the purchase of fast or prepared food increased?

- ◯ Yes
- ◯ No
- ◯ My consumption has not changed

* 8.3 During confinement, do you try to buy local products in these circumstances?

- Yes
- No, never
- Sometimes
- Do not know

9 In the kitchen

* 9.1 During confinement, what type of cooking techniques do you usually use?

between 1 and 4 choices

- Fried
- Roasting or baking
- Soups, stews or casseroles
- Papillote
- Stovetop
- Microwave oven
- Boiling and/or steam
- Other

* 9.2 During confinement, has the consumption of frying increased?

- Yes
- No
- My consumption has not changed

* 9.3 During confinement, does you spend more time cooking?

- Yes
- No
- Same as before

* 9.4 In your opinion, has the consumption of olive oil for cooking, including dressings, increased?

- Yes
- No
- Do not know

* 9.5 In your opinion, has the consumption of other cooking fats, including dressings, increased?

- Yes
- No
- Do not know

* 9.6 During confinement, how often do you rate your dishes with a good sofrito (oil, tomato, garlic, onion or leek, cooked on low heat)?

- Every day and with each dish
- Every day and with some dishes
- A few days of the week
- Never

* 9.7 In your opinion, do you think cooking with sofrito has increased?
  - Yes
  - No

10 Food consumption

* 10.1 During confinement, what amount of fresh fruit is consumed on a daily basis?
  - None, I do not like fruit
  - 1-2 pieces per day
  - 2-3 pieces per day
  - More than 3 pieces per day

* 10.2 In your opinion, do you think you eat more fresh fruit?
  - Yes
  - No

* 10.3 Do you normally consume fruit juices (1 or more on a daily basis) during confinement?
  - Natural
  - Packaged
  - Both interchangeably
  - No

* 10.4 During confinement, what is the amount of vegetables and vegetables do you eat on a daily basis?
  - None, I do not like vegetables
  - 1-2 portions per day (during lunch or dinner)
  - Minimum 2 (always part of my lunch and my dinner)

* 10.5 In your opinion, do you think you eat more vegetables?
  - Yes
  - No

* 10.6 During confinement, how many times a week do you eat legumes?
  - None, I do not like legumes
  - At least once a week
  - 2-3 times a week
  - More than three times a week

* 10.7 In your opinion, do you think you eat more legumes?
  - Yes
  - No

* 10.8 During confinement, what quantity of grains (bread, pasta, rice) do you eat on a daily basis?
  - None

- At least once a day
- Two or three times a day
- More than three times per day

* 10.9 In your opinion, do you think that you eat more grains during confinement?
- Yes
- No

* 10.10 In your opinion, do you think you eat more whole grains?
- Yes
- No

* 10.11 During confinement, what amount of potatoes (cooked, fried, oven, etc.) are consumed on a weekly basis?
- None
- At least once a week
- 2-3 times a week
- More than three times a week

* 10.12 In your opinion, do you think you eat more potatoes?
- Yes
- No

* 10.13 During the confinement, how many times a week do you eat nuts?
- None, I don't like nuts
- At least once a week
- 2-3 times a week
- More than three times a week

* 10.14 In your opinion, do you think you eat more nuts?
- Yes
- No

* 10.15 How many eggs do you consume on a weekly basis since confinement?
- None, do not like eggs
- At least once a week
- 2-3 times a week
- More than three times a week

* 10.16 In your opinion, do you think you eat more eggs?
- Yes
- No

* 10.17 During confinement, how many times a week do you eat fish/seafood?
- None, I do not like fish or seafood

- Every day
- At least once a week
- 2-3 times a week
- More than three times a week

* 10.18 In your opinion, do you think you eat more fish or seafood?

- Yes
- No

* 10.19 During confinement, how many times a week do you eat red meat (veal, lamb, etc.), sausages, hamburgers and sausages (including cured ham)?

- None, I don't consume these products
- Every day
- At least once a week
- 2-3 times a week
- More than three times a week

* 10.20 In your opinion, do you think you eat more red meat?

- Yes
- No

* 10.21 During confinement, how many times a week do you eat white meat (rabbit, chicken, turkey, etc.)?

- None, I don't consume these products
- Every day
- At least once a week
- 2-3 times a week
- More than three times a week

* 10.22 In your opinion, do you think you eat more white meat?

- Yes
- No

* 10.23 During confinement, how many times a day do you have dairy products (milk, cheese, curd and yoghurt)?

- None, I don't consume these products
- 1-2 servings per day
- 3-4 servings per day
- More than 4 servings per day
- Some times a week

* 10.24 In your opinion, do you think the consumption of dairy products has increased?

- Yes
- No

* 10.25 Please indicate the dairy products you had during the previous week:

- Ripened cheese
- Fresh cheeses
- Cream cheese
- Whole or skimmed natural yoghurts
- Sweetened yoghurts, with flavours, chocolate
- Fruit yoghurts
- Petit Suisse
- Curds
- I don't have dairy

* 10.26 During confinement, how much water do you drink on a daily basis?

- 5 or less glasses
- Between 6 and 8 glasses
- More than 8 glasses

* 10.27 In your opinion, do you drink more water? (as primary drink)

- Yes
- No

* 10.28 During the confinement, has your alcohol consumption been increased?

- Yes
- No

* 10.29 During confinement, please describe what kind of alcohol you consume in a weekly basis:

between 1 and 4 choices

- White, red, rosé or sparkling wine
- Beer
- Distillates
- Liqueurs
- I don't drink

* 10.30 During confinement, what amount of carbonate/sugar/energy drinks are consumed?

- Once a day
- 2 or more times per day
- Ever a week
- On a very sporadic basis
- Never

* 10.31 During the confinement, has your consumption of the carbonate drinks/sugar/energy beverages increased?

- Yes
- No

* 10.32 During the confinement, do you eat more homemade baked goods?

- Yes

- No
- No change

* 10.33 During the confinement, do you eat more industrial pastries?

- Yes
- No
- No change
